# Transcriptomic and Coexpression Network Analyses Revealed Pine *Chalcone Synthase* Genes Associated with Pine Wood Nematode Infection

**DOI:** 10.3390/ijms222011195

**Published:** 2021-10-17

**Authors:** Qiaoli Chen, Ruizhi Zhang, Danlei Li, Feng Wang

**Affiliations:** 1 Key Laboratory of Alien Forest Pests Monitoring and Control—Heilongjiang Province, School of Forestry, Northeast Forestry University, Harbin 150040, China; qiaolichen@nefu.edu.cn (Q.C.); zhangruizhi@nefu.edu.cn (R.Z.); danleili@nefu.edu.cn (D.L.); 2 Key Laboratory of Sustainable Forest Ecosystem Management—Ministry of Education, Northeast Forestry University, Harbin 150040, China

**Keywords:** *Bursaphelenchus xylophilus*, pine wilt disease, chalcone synthase, *Pinus thunbergii*, *P. massoniana*

## Abstract

Pine wood nematode (PWN) causes serious diseases in conifers, especially pine species. To investigate the transcriptomic profiles of genes involved in pine-PWN interactions, two different pine species, namely, *Pinus thunbergii* and *P. massoniana*, were selected for this study. Weighted gene coexpression network analysis (WGCNA) was used to determine the relationship between changes in gene expression and the PWN population after PWN infection. PWN infection negatively affects the expression of most genes in pine trees, including plant defense-related genes such as genes related to plant hormone signal transduction, plant-pathogen interactions, and the MAPK signaling pathway in plants. However, the expression of *chalcone synthase* genes and their related genes were proportional to the changes in nematode populations, and *chalcone synthase* genes were dominant within the coexpression module enriched by genes highly correlated with the nematode population. Many genes that were closely related to *chalcone synthase* genes in the module were related to flavonoid biosynthesis, flavone and flavonol biosynthesis, and phenylpropanoid biosynthesis. Pine trees could actively adjust their defense strategies in response to changes in the number of invasive PWNs, but the sustained expression of *chalcone synthase* genes should play an important role in the inhibition of PWN infection.

## 1. Introduction

Pine wood nematode (*Bursaphelenchus xylophilus*, PWN) causes pine wilt disease (PWD), mainly resulting in susceptible conifers, in particular *Pinus* spp., to wilt rapidly and die [1]. PWN requires an insect vector for local dispersal and infection [2]. The nematode can be transmitted when adult insects feed on the phloem of young twigs on healthy trees for sexual maturation [3,4] or during female oviposition into dying trees or cutting waste [5,6]. Once a PWN enters a tree, it can migrate through cortical and xylem axial and radial resin canals in pine stems and feed on plant tissues, which causes a series of physiological and biochemical changes, leading to water deficiency and tree mortality [1,7,8].

PWN is one of the main threats to conifer forests worldwide. The nematode is responsible for millions in losses every year and is considered a quarantine organism by the European and Mediterranean Plant Protection Organization. After PWN spread to East Asia, PWD became the most serious disease in pine forests due to changes in conditions such as hosts, vector insects, and the environment [9,10,11,12,13], and PWN has also been found in Mexico [14], Portugal [15], and Spain [16,17]. However, PWN occurs only sporadically in North America, where it originates [18,19].

PWD has been studied extensively for its pathogenesis and prevention since the 1970s [1]. However, due to its high morbidity rate, concealed occurrence, various modes of transmission, and wide distribution range, there is still a lack of effective control measures, and there is a trend of further spread. At present, elucidating the pathogenic mechanism of PWN and selecting and applying resistant pines are important research tasks for the development of control strategies for PWD. However, there remains a lack of adequate understanding of the molecular basis of the interactions between PWN and its host plants [20].

PWN is known to infest a large range of pine species, but different species show different levels of susceptibility. Within a species, it is possible to identify trees with contrasting responses to PWN infection [21]. Several studies have focused on characterizing the transcriptomic profiles to understand the differences in defense mechanisms against PWN infection in species of *Pinus* with different susceptibilities to PWD [21,22,23,24,25]. However, due to the lack of genomic information and the complexity of the disease, the molecular response of pines to PWN is still not well understood. Further studies are required to understand the defensive mechanisms of tolerant pines in response to PWN infection.

When a plant is infected by a pathogen, a series of changes, including changes in morphology, physiology, biochemistry, and molecular biology, occur in the plant [26,27]. Plant resistance to pathogens is dependent on the morphology, nutritional quality, and accumulation of secondary metabolites [26]. In this study, we used artificial inoculation with PWN to analyze the changes in gene expression in two different pine species, namely, *Pinus thunbergii* and *P. massoniana*, in response to PWN infection. Weighted gene coexpression network analysis (WGCNA) was used to explore the complex relationships between genes and phenotypes, which helped in determining the main functions of genes in the modules related to defensive mechanisms in response to PWN infection [28]. The objective of this study was to investigate the physiological response of host pine trees to PWN infection and to provide references for the study of the pathogenicity and resistance mechanism of the interaction between these organisms.

## 2. Results

### 2.1. Changes in Pines and Nematode Populations after PWN Inoculation

**Figure 1:**
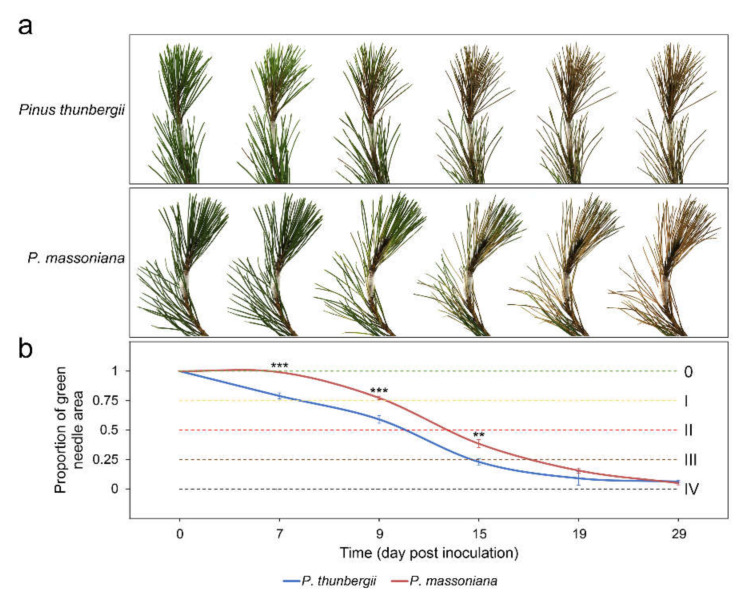
Changes in pines inoculated with pine wood nematodes. (**a**) Symptoms of pines after inoculation; (**b**) change in the color of the pine needles. 0: healthy; Ⅰ: a quarter of the needles were chlorotic; Ⅱ: half of the needles were chlorotic, turned brown and died, and the branch tip was deformed and bent; Ⅲ: three-quarters of the needles were chlorotic, turned brown and died, and the branch tip sagged; Ⅳ: all of the needles were chlorotic, turned brown and died, and the whole plant wilted. Error bars indicate the standard deviation (*** *p* value < 0.001, ** *p* value < 0.01, *P. massoniana* test *P. thunbergii*).

There were no obvious symptoms for all the control pines, however, *P. thunbergii* and *P. massoniana* reacted differently after inoculation with PWN (Figure 1). For *P. thunbergii*, symptoms began to appear at 7 days post inoculation (dpi), when approximately a quarter of the needles of the inoculated pines were chlorotic. All the inoculated pines exhibited symptoms at 15 dpi, when approximately three-quarters of the needles of the inoculated pines were chlorotic. The whole inoculated pine plants withered and died by 19 dpi. The period from onset to the browning of the needles and the dry death of the stem segments lasted 12 days. For *P. massoniana*, symptoms began to appear at 9 dpi, when approximately a quarter of the needles of the inoculated pines were chlorotic. All the inoculated pines exhibited symptoms at 19 dpi, when approximately three-quarters of the needles of the inoculated pines were chlorotic. The whole inoculated pine plants withered and died by 29 dpi. The period from onset to the browning of the needles and the dry death of the stem segments lasted 20 days. These results suggested that *P. thunbergii* was more susceptible to PWD than *P. massoniana*.

**Figure 2:**
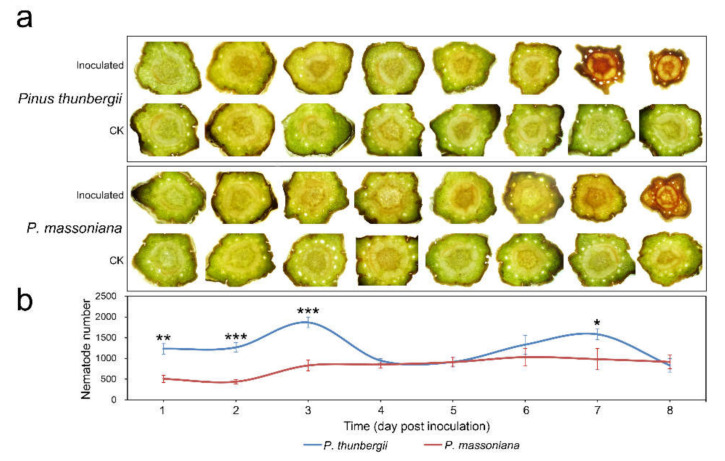
Plant tissue section observation and changes in nematode populations after PWN inoculation. (**a**) Changes in segments 1 cm below inoculation sites; (**b**) changes in nematode populations. Error bars indicate the standard deviation (*** *p* value < 0.001; ** *p* value < 0.01; * *p* value < 0.05; *P. thunbergii* test *P. massoniana*).

The plant tissue section observation indicated that the stem segments near the inoculation sites dried up and shrunk first. The changes in segments 1 cm below the inoculation sites are shown in Figure 2a. The wilting of the stem of *P. massoniana* occurred later than that of *P. thunbergii*. Upon comparing the changes in the population of PWN in the two pine species after inoculation, it was found that the PWN population in *P. thunbergii* from 1 dpi to 3 dpi was higher than that in *P. massoniana* (Figure 2b), indicating that *P. thunbergii* was more likely to be infected by PWN.

### 2.2. Transcriptome Sequencing

Based on the finding that *P. thunbergii* and *P. massoniana* showed different symptoms in response to PWN infection and that the populations of PWN showed different variations, we wondered whether the changes in nematode populations were related to the expression of defense-related genes in pine trees. Therefore, we analyzed the changes in gene expression in the two pine species after inoculation with PWN. For each pine, a 2 cm-long segment of the stem was taken from 1 cm below the inoculation site. Since this segment of *P. thunbergii* wilted at 7 dpi, we procured the samples from 1 dpi to 6 dpi. To characterize the gene transcription patterns, inoculated samples of the two different pine species at six different time points (1 dpi, 2 dpi, 3 dpi, 4 dpi, 5 dpi, and 6 dpi) and two control samples at 1 dpi (one for each pine) were selected.

In total, 14 libraries were constructed and sequenced. An average of 4.40 Gb was obtained for each sample (Table S1). The dataset was deposited in the Sequence Read Archive (SRA, BioProject ID: PRJNA744619). Then, we assembled all the samples of *P. massoniana* together and obtained 49,175 unigenes, and the total length, average length, N50, and GC content of the unigenes were 47,688,965 bp, 969 bp, 1676 bp, and 43.19%, respectively. The unigenes were then compared to functional databases for annotation. In total, 33,883 (nucleotide sequence database, NT: 68.90%), 34,299 (non-redundant protein database, NR: 69.75%), 23,834 (SwissProt: 48.47%), 25,310 (Kyoto Encyclopedia of Genes and Genomes, KEGG: 51.47%), 15,115 (Cluster of Orthologous Groups of proteins, COG: 30.74%), and 13,241 (Gene Ontology, GO: 26.93%) unigenes received functional annotations. Moreover, we assembled all the samples of *P. thunbergii* together and obtained 47,333 unigenes, and the total length, average length, N50, and GC content of the unigenes were 46,070,766 bp, 973 bp, 1688 bp, and 43.00%, respectively. In total, 32,617 (NT: 68.91%), 32,832 (NR: 69.36%), 22,620 (SwissProt: 47.79%), 24,026 (KEGG: 50.76%), 14,116 (COG: 29.82%), and 12,353 (GO: 26.10%) unigenes received functional annotations. Then, clean reads were mapped to unigenes and gene expression levels for each sample were calculate.

### 2.3. WGCNA Revealed Modules Related to the Nematode Population

**Figure 3:**
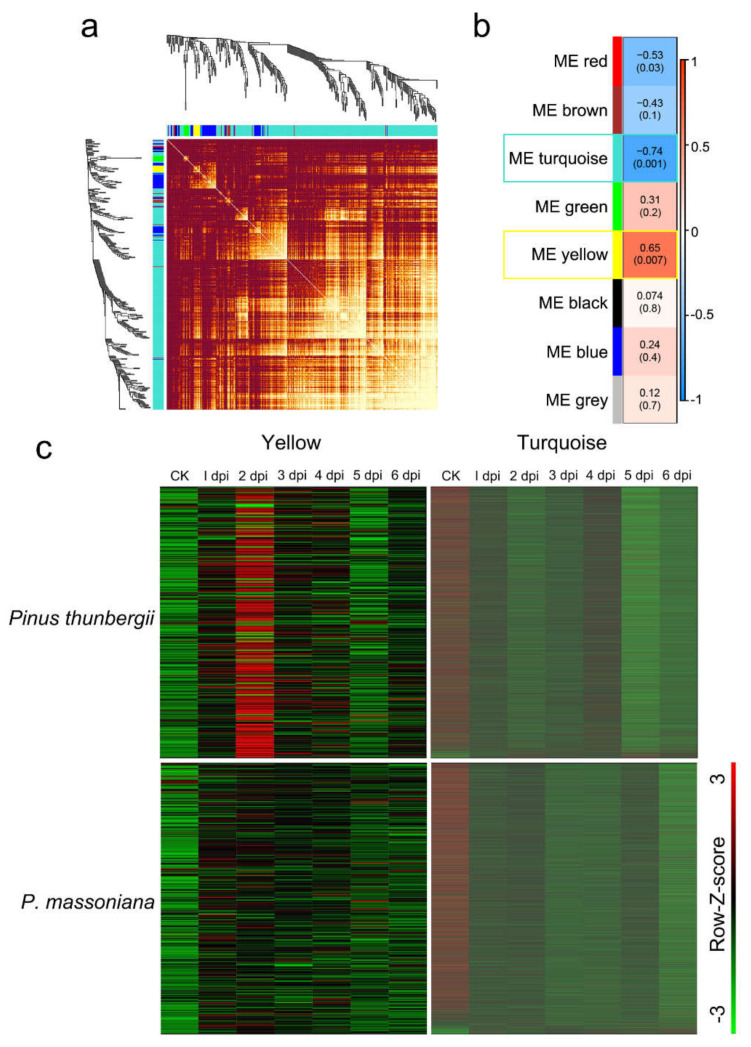
WGCNA revealed modules closely related to the nematode population after inoculation. (**a**) Visualizing the gene network using a heatmap plot (400 genes were randomly selected). The heatmap depicts the topological overlap matrix among all genes in the analysis. Dark red color represents low overlap and progressively brighter color represents higher overlap. Blocks of brighter colors along the diagonal are the modules. The gene dendrogram and module assignment are also shown along the left side and the top. (**b**) Associations for module eigengene and nematode population. Each cell contains the corresponding correlation and *p* value. The table is color coded by correlation according to the color legend. (**c**) Heatmap of module gene expression (rows) across the samples (columns) for yellow and turquoise modules.

After determining the gene expression patterns of ortholog genes from the two different pines at different time points after PWN infection, WGCNA was applied to obtain gene sets exhibiting strong correlations with changes in the nematode population. To eliminate noise from genes that were not expressed or expressed at low levels, we filtered probes with median transcripts per kilobase of exon model per million mapped reads (TPM) levels that did not exceed 1. Then the expression values of 11,861 genes in 14 transcriptomes were obtained and used to construct the coexpression module with WGCNA package tools. Cluster analysis was performed on these samples to ensure that there were no obvious outliers (Figure S1). We chose a power of 9, which was the lowest power at which the scale-free topology fit index curve flattened out upon reaching a high value (in this case, approximately 0.76; Figure S2), to construct coexpression modules. Seven distinct gene coexpression modules were identified (Figure S3) to screen out genes with similar expression patterns and the number of genes in each module is shown in Table S2 (grey was reserved for unassigned genes). The interactions of the seven coexpression modules were analyzed, and the gene network was visualized by plotting a heatmap (Figure 3a). Each set of highly correlated genes corresponded to a branch of the tree. There was often a high topological overlap between genes in the same module.

The module eigengene (ME) is defined as the first principal component of a given module. It can be considered representative of the gene expression profiles in a module. Modules with common expression pattern interactions among the coexpression modules that were associated with particular traits were identified based on the correlation between the ME and the trait (Figure 3b). The analysis revealed that the turquoise module was significantly negatively associated with changes in the nematode population (correlation value, cor = −0.74, *p*-value = 0.001) and that the yellow module was significantly positively associated with changes in the nematode population (cor = 0.65, *p*-value = 0.007).

To further analyze the differences in the expression of nematode population-related genes in the two pines at different time points, the expression patterns of all genes in the selected modules were compared. There were 9275 genes in the turquoise module, accounting for 78.20% of the total analyzed genes. These results indicated that after PWN infection, an increase in the number of nematodes affected the expression of most genes in pine trees. There were 278 genes in the yellow module, accounting for only 2.34% of the total analyzed genes. Changes in the expression of these genes in the yellow module were proportional to changes in the nematode population, suggesting that they might be involved in inhibiting the increase in nematode populations.

**Figure 4:**
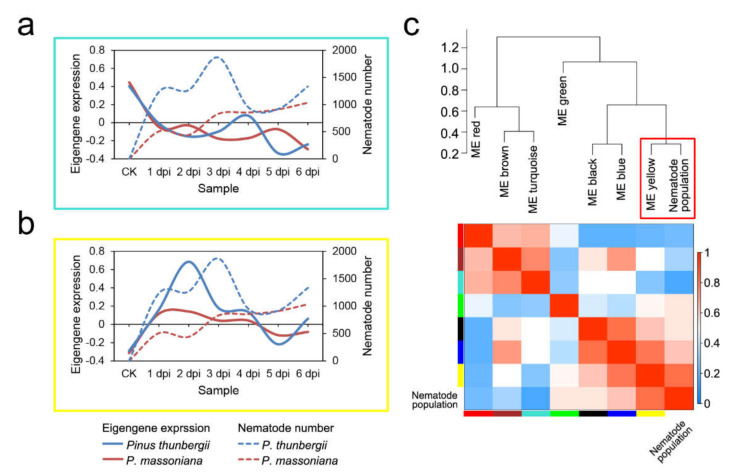
Module eigengene expression pattern and correlation. (**a**) Comparison of eigengene expression (y-axis) in the turquoise module across the samples (x-axis); (**b**) comparison of eigengene expression in the yellow module across the samples; (**c**) hierarchical clustering dendrogram and heatmap of the correlated eigengenes and nematode population. ME, module eigengene.

For each of the selected modules, an expanded view of the expression of all genes in the module was compared with the ME expression of the module across all samples. The ME values of the turquoise module and the yellow module were compared across the samples (Figure 4a,b). The ME took on low values in arrays where many module genes were underexpressed (Figure 3c, green color in the heatmap). The ME took on high values in arrays where many module genes were overexpressed (Figure 3c, red in the heatmap). The results indicated that the genes in the turquoise module were downregulated in both pine species after PWN infection and the genes in the yellow module were the most obviously upregulated at 2 dpi in *P. thunbergia*. To study the relationships among the identified modules, the eigengenes were used as representative profiles, and eigengene correlation was used to quantify module similarity. The trait that determined the changes in the nematode population was added to the eigengenes to determine how this trait fit into the eigengene network. The dendrogram indicated that the yellow module had the highest association with the nematode population (Figure 4c).

### 2.4. Functional and Pathway Enrichment Analyses of Genes in a Nematode Population-Related Module

GO enrichment and KEGG enrichment analyses were performed on the genes in the turquoise and yellow modules to identify the functions of these genes and the pathways that might be involved. GO enrichment analysis revealed that genes in the turquoise and yellow modules were mainly enriched in catalytic activity (GO:0003824) and binding (GO:0005488) in the molecular function category; in cell (GO:0005623), cell part (GO:0044464), and intracellular (GO:0005622) in the cellular component category; and metabolic process (GO:0008152) in the biological process category. In addition, many genes in the turquoise module were enriched in cellular nitrogen compound metabolic process (GO:0034641), gene expression (GO:0010467), localization (GO:0051179), heterocycle metabolic process (GO:0046483), adenyl ribonucleotide binding (GO:0032559), and nucleic acid binding (GO:0003676, Table S3). However, many genes in the yellow module were enriched in oxidoreductase activity (GO:0016491), transition metal ion binding (GO:0046914), organic acid metabolic process (GO:0006082), biosynthetic process (GO:0009058), carboxylic acid metabolic process (GO:0019752), and oxoacid metabolic process (GO:0043436, Table S4).

Based on the KEGG enrichment analysis, genes in the turquoise and yellow modules were mainly enriched in metabolic pathways (ko01100) and biosynthesis of secondary metabolites (ko01110). In addition, many genes in the turquoise module were enriched in the ribosome (ko03010), spliceosome (ko03040), plant hormone signal transduction (ko04075), protein processing in endoplasmic reticulum (ko04141), carbon metabolism (ko01200), plant–pathogen interaction (ko04626), RNA transport (ko03013), and MAPK signaling pathway plant (ko04016, Table S5). However, many genes in the yellow module were enriched in flavonoid biosynthesis (ko00941), biosynthesis of amino acids (ko01230), and phenylpropanoid biosynthesis (ko00940, Table S6).

### 2.5. Finding Genes with High Gene Significance and High Intramodular Connectivity in Interesting Modules

**Figure 5:**
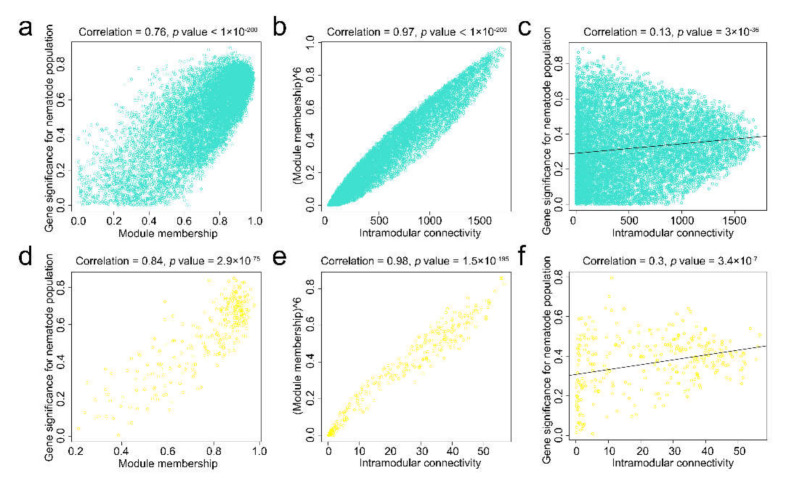
Relationship between gene significance (GS), module membership (MM), and intramodular connectivity (IC). (**a**) GS for the nematode population (y-axis) vs. MM (x-axis) for the turquoise module; (**b**) MM raised to power 6 (y-axis) vs. IC (x-axis) for the turquoise module; (**c**) GS (y-axis) vs. IC (x-axis) for the turquoise module; (**d**) GS for the nematode population vs. MM for the yellow module; (**e**) MM raised to power 6 vs. IC for the yellow module; (**f**) GS vs. IC for the yellow module.

To quantify the similarity of genes in the turquoise module and the yellow module, the associations of individual genes with nematode populations were quantified by defining gene significance (GS) as the correlation between the gene and the trait, and a quantitative measure of module membership (MM) was also defined as the correlation of the ME and the gene expression profile for the module. A scatterplot of GS vs. MM in the turquoise module and the yellow module was plotted, and GS and MM were highly correlated, illustrating that genes highly significantly associated with a trait were often also the most important (central) elements of modules associated with the trait (Figure 5a,d).

The intramodular connectivity (IC) value was defined only for the genes within a given module. The IC measures how connected or coexpressed a given gene is with respect to the genes of a particular module and may be interpreted as a measure of MM. The IC for each gene in the turquoise module and the yellow module was calculated. Genes with the top 30 highest IC values were considered intramodular hub genes in this study (Tables S7 and S8). After raising the MM to a power of 6, it was found to be highly correlated with the IC (Figure 5b,e). Highly connected intramodular hub genes tend to have high MM values in the respective module. A GS vs. IC plot was generated. For both the turquoise module and the yellow module, we observed that intramodular hub genes tended to have high GS (Figure 5c,f).

Of the 30 hub genes in the turquoise module, 10 were enriched in 15 pathways (Table S9). Among them, most genes were enriched in plant hormone signal transduction (ko04075), followed by metabolic pathways (ko01100), RNA degradation (ko03018), and peroxisomes (ko04146). Moreover, there was one gene enriched in the MAPK signaling pathway—plant (ko04016). These results suggested that the dominant genes in the turquoise module are mostly related to signal transduction. Of the top 30 genes with the highest negative value of GS for the nematode population in the turquoise module (Table S10), 7 were enriched in 12 pathways (Table S11). Among them, most genes were enriched in metabolic pathways (ko01100), followed by biosynthesis of secondary metabolites (ko01110).

Of the 30 hub genes in the yellow module, 18 were enriched in 22 pathways (Table S12). Among them, most genes were enriched in metabolic pathways (ko01100) and biosynthesis of secondary metabolites (ko01110), followed by flavonoid biosynthesis (ko00941). In addition, 5 of the hub genes were ortholog genes of *chalcone synthase*. Of the top 30 genes with the highest GS for the nematode population in the yellow module (Table S13), 15 were enriched in 24 pathways (Table S14). Among them, most genes were enriched in metabolic pathways (ko01100), followed by biosynthesis of secondary metabolites (ko01110) and flavonoid biosynthesis (ko00941). These results suggested that in the yellow module, the gene function of the dominant genes was consistent with that of genes highly correlated with the population of nematodes. Six genes belonged to both the top 30 genes with the highest value of IC (hub genes) and the top 30 genes with the highest GS. Three of those six genes were enriched in flavonoid biosynthesis, including an ortholog gene of *chalcone synthase*.

### 2.6. Expression Profiles and Coexpression Network of Candidate Genes

**Figure 6:**
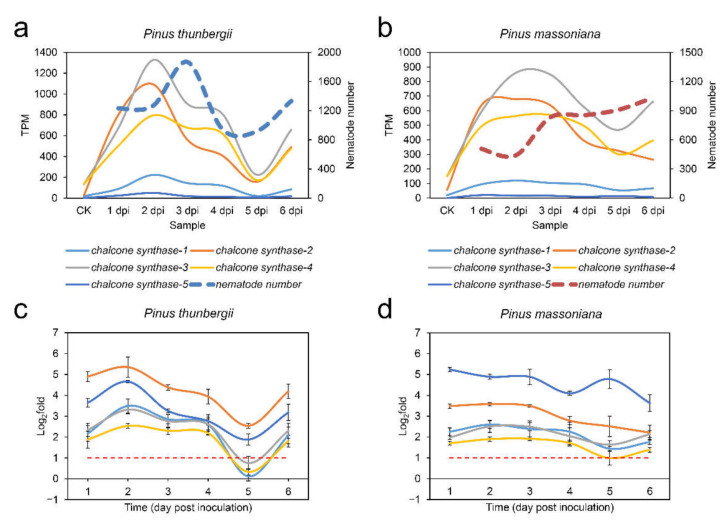
Expression patterns of *chalcone synthase* genes at different time points. (a) Expression levels for *chalcone synthase* genes based on per kilobase of exon model per million mapped reads (TPM) in *Pinus thunbergii*; (b) expression levels for *chalcone synthase* genes based on TPM in *P. massoniana*; (c) changes in expression levels verified by real-time quantitative PCR (RT-qPCR) for *chalcone synthase* genes in *P. thunbergii*; (d) changes in expression levels verified by RT-qPCR for *chalcone synthase* genes in *P. massoniana*. The data are given as the mean or mean with standard deviation.

The above results indicated that *chalcone synthase* genes were dominant in the yellow module, and their expression was positively correlated with the population of nematodes. The five *chalcone synthase* genes mentioned above that were hub genes in the yellow module were selected for further study. Their expression levels at different time points indicated that the expression of these genes in *P. thunbergii* was not significantly different from that of CK at 5 dpi, although the expression of these genes was upregulated at other time points, especially at 2 dpi; furthermore, the expression of these genes was upregulated at all time points in *P. massoniana* (Figure 6a,b). Their expression levels at different time points were further verified by Real-time quantitative PCR (RT-qPCR, Figure 6c,d). The results indicated that these *chalcone synthase* genes showed no significant change or were upregulated to a lesser degree than at other time points in *P. thunbergii* at 5 dpi.

**Figure 7:**
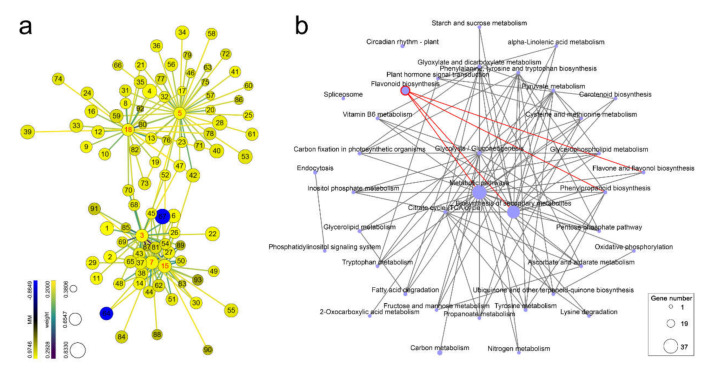
Gene network for *chalcone synthase* genes in the yellow module. (**a**) Coexpression network for *chalcone synthase* genes (weight value > 0.2, detailed in Table S15). Prefuse force directed layout was applied based on the weight value between two genes. The size of the nodes represents the gene significance for the nematode population (from 0.3806 to 0.8330). The color of the nodes represents module membership (from −0.8649 to 0.9746). The colors of the lines represent the weight value between two genes (from 0.2000 to 0.2928). The labels on the nodes are based on intramodular connectivity (from 6.1505 to 56.6806). The *chalcone synthase* genes are highlighted with red labels; (**b**) KEGG enrichment network. The nodes represent KEGG pathways, and the node size represents the number of genes enriched in the pathway by KEGG enrichment analysis. The pathways that *chalcone synthase* genes were enriched in are highlighted with red borders.

For each of the five *chalcone synthase* genes, genes highly correlated with them in the yellow module were screened. The genes in the yellow module whose weight values with *chalcone synthase* genes were greater than 0.2 were selected, and their network data were exported to Cytoscape by Prefuse Force Directed Layout based on the weight values between two genes. The whole network contained 163 regulatory relationships of 93 genes (Figure 7a, details can be found in Table S15). Among the 93 genes in the yellow module, 47 were enriched in 40 different pathways (Table S16). Among them, most genes were enriched in metabolic pathways (ko01100) and biosynthesis of secondary metabolites (ko01110), followed by flavonoid biosynthesis (ko00941). There were three *chalcone synthase* genes (No. 3, *chalcone synthase* 1; No. 7, *chalcone synthase* 3; and No. 15, *chalcone synthase* 4) enriched in flavonoid biosynthesis. The correlation among the enriched pathways was studied, and it was found that flavonoid biosynthesis was related to metabolic pathways, biosynthesis of secondary metabolites, flavone and flavonol biosynthesis (ko00944), and phenylpropanoid biosynthesis (ko00940, Figure 7b). Therefore, after PWN invaded pine trees, its population size was proportional to the expression of several *chalcone synthase* genes in pine trees, and *chalcone synthase* genes were mainly involved in flavonoid biosynthesis, which is related to flavone and flavonol biosynthesis, and phenylpropanoid biosynthesis and they might affect the population of PWN in pine trees.

## 3. Discussion

In nature, plants are exposed to various biotic and abiotic stresses. Under stress conditions, plants express a number of genes as part of their defense [29]. Among these genes, *chalcone synthase* is quite commonly induced in different plant species under different forms of stress. Chalcone synthase is the key enzyme for the synthesis of flavonoids and is also the rate-limiting enzyme [30,31]. As a secondary metabolite synthesized via the phenylpropanoid and polyketide pathways, flavonoids are widely distributed in plants and play an important role. Under stress conditions, such as ultraviolet radiation and bacterial or fungal infection, plants promote the expression of chalcone synthase, activate the phenylalanine biosynthesis pathway, and promote the accumulation of flavonoids or isoflavones, resulting in the production of compounds that have, for example, antimicrobial activity (phytoalexins), insecticidal activity, and antioxidant activity or quench ultraviolet light directly or indirectly, thereby protecting themselves [31,32,33]. In plants, chalcone synthase may always be present in the cells but is only activated under certain conditions [31].

In this study, we found that most genes, including plant defense-related genes such as genes related to plant hormone signal transduction, plant–pathogen interactions, and the MAPK signaling pathway in plants, were downregulated in the two pine species after PWN infection, and the changes in their expression were negatively correlated with the changes in nematode populations. These results suggested that PWN infection negatively affects the expression of most genes in pine trees. On the other hand, the expression of many *chalcone synthase* genes and their related genes continued to increase after PWN infection, and the changes in the expression of these genes were proportional to the changes in nematode populations. These results suggested that the expression of *chalcone synthase* genes and their related genes plays an important role in the resistance of pine trees to PWN infection.

We also found that the population of PWN did not continue to increase after the pine trees were infected. The number of PWNs in *P. thunbergii* was significantly higher than that in *P. massoniana* in the first three days. Correspondingly, the expression of *chalcone synthase* genes was upregulated significantly in both pine species, but the expression level of most *chalcone synthase* genes in *P. thunbergii* was slightly higher than that in *P. massoniana*. This indicated that as the number of nematodes that entered pine trees increased, the expression of *chalcone synthase* genes also increased. From 4 dpi to 6 dpi, there was no significant difference in the number of PWNs in the two pine species, but during this period, the number of PWNs in *P. thunbergii* showed a decline. This might have been due to the continuous high expression of *chalcone synthase* genes, resulting in the gradual death of a large number of nematodes. The expression of *chalcone synthase* genes decreased with decreasing PWN population size in *P. thunbergii*. This suggested that pine trees actively adjust their defense strategies in response to changes in the number of invasive PWNs. Therefore, although the expression levels of most *chalcone synthase* genes were higher, the more susceptible pine species, *P. thunbergii*, could not inhibit PWN infection in a timely manner but could inhibit PWN population growth at some point.

In contrast to the increase followed by a decrease in the population of PWN in *P. thunbergii*, the population of PWN in *P. massoniana* showed a moderate change from 3 dpi. These results suggested that the less susceptible pine species, *P. massoniana*, could effectively inhibit the population growth of PWN, which was related to the continuous expression of *chalcone synthase* genes. Chalcone synthase can help plants produce additional flavonoids, isoflavonoid-type phytoalexins, and other related metabolites to protect them against stress [34,35]. Phytoalexins are antimicrobial metabolites produced by plants in response to microbial attack (or biotic and abiotic elicitors) [34]. The accumulation of flavonoids and isoflavonoids in response to pathogen attack has been observed in many plant species, and their importance as antimicrobial phytoalexins is well established [34,36,37]. Therefore, sustained expression of *chalcone synthase* genes should play an important role in the inhibition of PWN infection.

In this study, we found that *chalcone synthase* genes were dominant within the module enriched by genes highly correlated with nematode populations. The closely related genes in the module were highly enriched in flavonoid biosynthesis, flavone and flavonol biosynthesis, and phenylpropanoid biosynthesis, indicating that pine trees might inhibit PWN invasion by activating *chalcone synthase* expression to accumulate phytoalexins. Therefore, inducing the expression of *chalcone synthase* genes may improve the resistance of pine to PWN, but the underlying mechanism and activation pathway need to be further studied.

## 4. Materials and Methods

### 4.1. Biological Material and Pine Wood Nematode Inoculation

PWN (collected in Ningbo, Zhejiang Province, China) was cultured on *Botrytis cinerea* at 25 °C in the dark. A Baermann funnel was used to extract the nematodes (male, female, and juvenile nematodes mixed together at a ratio of 1:1:2). Inoculation with PWN was conducted following the method described in our previous study [38,39]. The seedlings used were derived from seeds and maintained under natural environmental conditions in a greenhouse. Three-year-old *P. thunbergii* or *P. massoniana* seedlings were inoculated with a water suspension of cultured nematodes (10,000 nematodes in 500 μL of ddH_2_O/seedling) for treatment or inoculated with ddH_2_O (500 μL/seedling) as a control.

Five biological replicates were set for each treatment, and three groups were set for each biological replicate. For group one, the seedlings were cut into pieces separately, and a Baermann funnel was used to collect the nematodes to calculate changes in nematode populations. For group two, the seedlings were cut and sliced for plant tissue section observation. For group three, a 2 cm-long segment of stem 1 cm below the inoculation site for each seeding was cut and put into liquid nitrogen immediately in the field and stored at −80 °C for RNA extraction. Samples were collected everyday post inoculation.

### 4.2. RNA Extraction, cDNA Synthesis, Library Preparation and Sequencing

Total RNA from each sample of inoculated trees and control trees was separately extracted using the RN38 EASYSpin Plus Plant RNA Kit (Aidlab Biotech, Beijing, China) following the manufacturer’s procedure [24]. The total RNA quantity and purity were analyzed with a Bioanalyzer 2100 and RNA 1000 Nano LabChip Kit (Agilent Technologies, Santa Clara, CA, USA) with RIN number > 7.0. Poly(A) RNA was purified from total RNA using poly-T oligo-attached magnetic beads using two rounds of purification. Following purification, the mRNA was fragmented into small pieces using divalent cations under elevated temperature. Then, the cleaved RNA fragments were reverse transcribed to create the final cDNA library in accordance with the protocol for the mRNASeq sample preparation kit (Illumina, San Diego, CA, USA), and the average insert size for the paired-end libraries was 300 bp (±50 bp). Then, paired-end sequencing was performed on the Illumina HiSeq 4000 platform (LC Sciences, Houston, TX, USA) following the vendor’s recommended protocol.

### 4.3. De Novo Assembly, Unigene Annotation and Functional Classification

First, in-house Cutadapt [40] and Perl scripts were used to remove the reads that contained adaptor contamination, low-quality bases and undetermined bases. Then, the sequence quality was verified using FastQC (version 0.11.9, http://www.bioinformatics.babraham.ac.uk/projects/fastqc, accessed on 5 August 2019), including the Q20, Q30, and GC content of the clean data. All downstream analyses were based on clean data of high quality. De novo assembly of the transcriptome was performed with Trinity 2.4.0 [41]. Trinity groups transcripts into clusters based on shared sequence content. The longest transcript in the cluster was chosen as the unigene. All assembled unigenes were aligned against NT (http://www.ncbi.nlm.nih.gov, accessed on 11 May 2021), NR (http://www.ncbi.nlm.nih.gov, accessed on 12 May 2021), GO (http://www.geneontology.org, accessed on 9 May 2019), SwissProt (http://www.expasy.ch/sprot, accessed on 11 May 2019), COG (http://www.ncbi.nlm.nih.gov/COG, accessed on 10 May 2019), and KEGG (http://www.genome.jp/kegg, accessed on 13 May 2019) databases using DIAMOND [42] with a threshold e-value < 0.00001. Salmon [43] was used to determine the expression level of unigenes by calculating TPM [44].

### 4.4. Weighted Gene Coexpression Network Analysis

WGCNA (https://horvath.genetics.ucla.edu/html/CoexpressionNetwork, accessed on 7 August 2018) was used to explore the complex relationships between genes and phenotypes [28]. The appropriate power value was determined when the degree of independence was over 0.8. The minimum number of genes was set as 30 for high reliability of the results. Module–trait associations were estimated using the correlation between the ME and the trait. IC was calculated for each gene by summing the connection strengths with other module genes and dividing this number by the maximum IC. The IC value was defined only for the genes within a given module. IC measures how connected or coexpressed a given gene is with respect to the genes of a particular module. For each expression profile, GS was calculated as the absolute value of the Pearson correlation between the expression profile and each trait. MM was defined as the Pearson correlation of the expression profile and each ME. Network depictions were constructed by Cytoscape software (version 3.7.1, https://cytoscape.org/, accessed on 25 February 2019) [45].

### 4.5. Real-Time Quantitative PCR Analysis

RT-qPCR was performed with the GoTaq 2-Step RT-qPCR System Kit (Promega, Madison, WI, USA, catalogue number: A6010) and the Stratagene Mx3000P qPCR system (Agilent Technologies, Santa Clara, CA, USA) to validate the transcript levels of the genes. *Ubiquitin* was used as the internal control. All primers used in this study are listed in Table S17. The normalization of the data was performed according to the instructions for the GoTaq 2-Step RT-qPCR System Kit and by the 2^−ΔΔCT^ method [46]. The experiments were repeated three times. Significance was determined by Student’s *t*-test.

## 5. Conclusions

*Chalcone synthase* genes dominated within the coexpression module and were enriched by genes highly correlated with nematode populations. Many genes that were closely related to *chalcone synthase* genes in the module were related to flavonoid biosynthesis, flavone and flavonol biosynthesis, and phenylpropanoid biosynthesis. Sustained expression of *chalcone synthase* genes should play an important role in the inhibition of PWN infection.

## Data Availability

The datasets are available in the Sequence Read Archive (BioProject ID: PRJNA744619).

## Supplementary Materials

The following are available online at https://www.mdpi.com/article/10.3390/ijms222011195/s1.
