# Supplementary material for: Transcriptomic and Coexpression Network Analyses Revealed Pine Chalcone Synthase Genes Associated with Pine Wood Nematode Infection
Source: Int J Mol Sci. 2021 Oct 17;22(20):11195. doi: 10.3390/ijms222011195 (PMC8540587; doi:10.3390/ijms222011195)
Supplement: Supplementary file 1 [file ijms-22-11195-s001.zip › ijms-1417414-supplementary.pdf]

Transcriptomic and Coexpression Network Analyses Revealed Pine *Chalcone Synthase* Genes Associated with Pine Wood Nematode Infection

Qiaoli Chen [1,2†], Ruizhi Zhang [1†], Danlei Li [1,2], Feng Wang [1,2*]

[1] Key Laboratory of Alien Forest Pests Monitoring and Control-Heilongjiang Province, School of Forestry, Northeast Forestry University, Harbin 150040, China

[2] Key Laboratory of Sustainable Forest Ecosystem Management-Ministry of Education, Northeast Forestry University, Harbin 150040, China

[*] Correspondence: fengwang@nefu.edu.cn (F.W.)

[†] These authors contributed equally to this work.

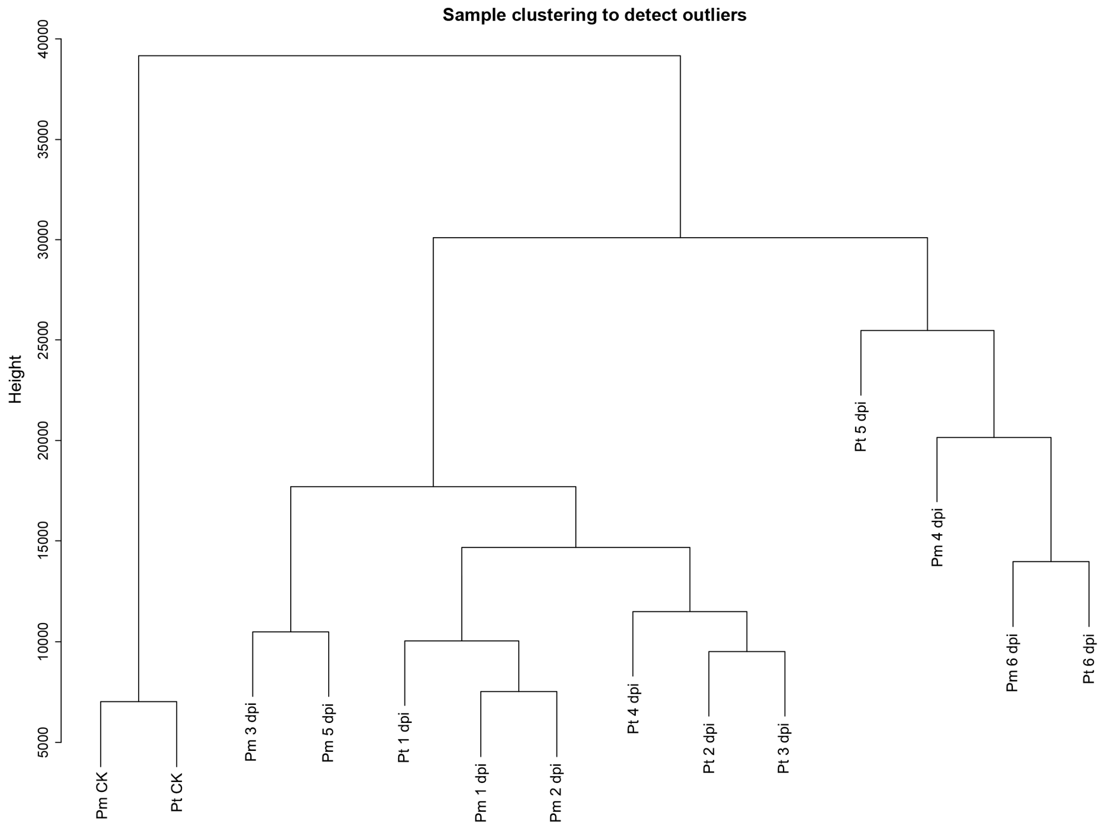

Figure S1. Sample clustering to detect outliers.

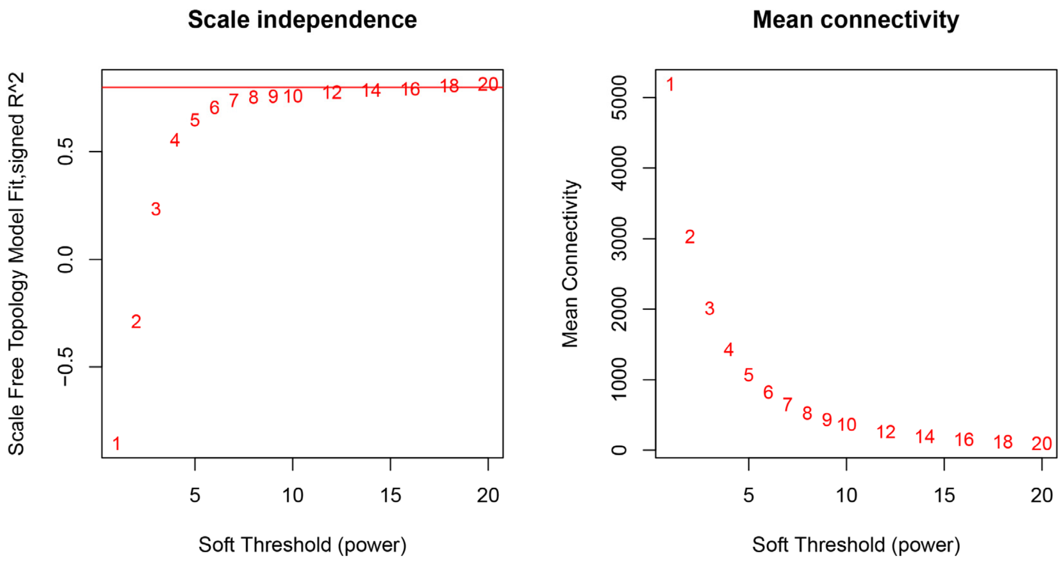

Figure S2. Soft power selection.

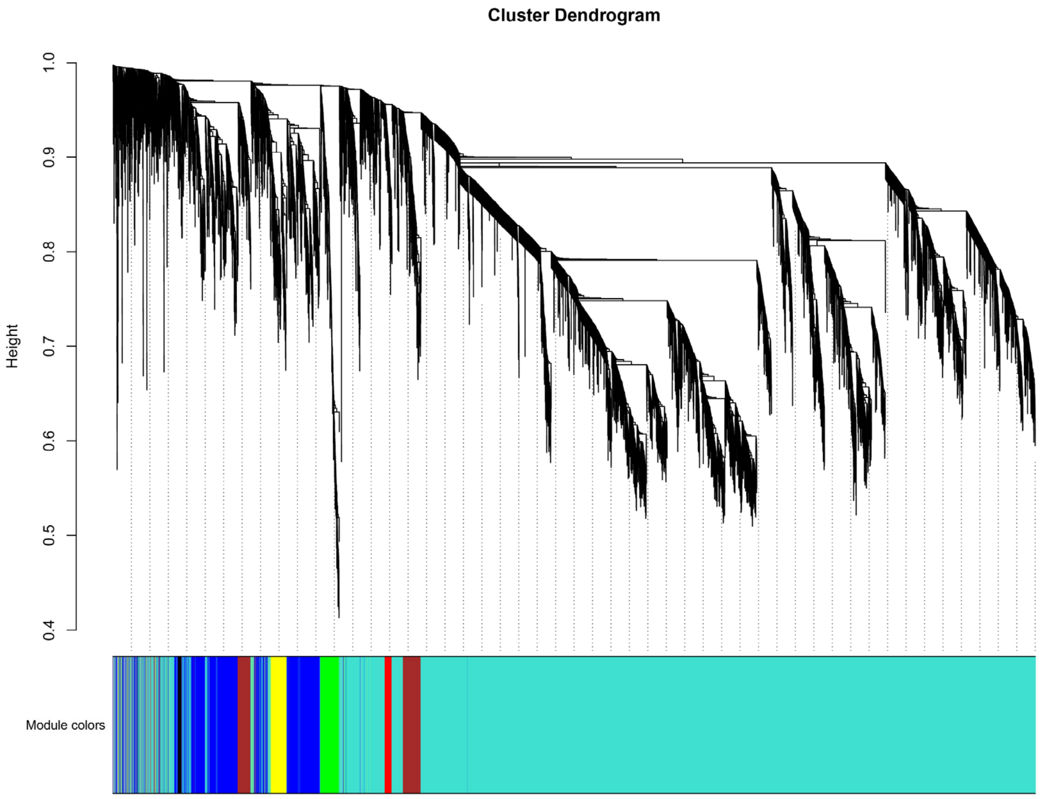

Figure S3. Cluster dendrogram.

Table S1. Quality statistics of filtered reads.

Sample	Total raw reads (Mb)	Total clean reads (Mb)	Total clean bases (Gb)	Clean reads Q20 (%)	Clean reads Q30 (%)	Clean reads ratio (%)
Pt 1 dpi	34.16	29.44	4.41	98.79	96.29	86.19
Pt 2 dpi	34.85	29.79	4.47	98.86	96.33	85.49
Pt 3 dpi	33.25	28.55	4.28	98.88	96.24	85.87
Pt 4 dpi	32.98	28.16	4.22	98.90	95.89	85.40
Pt 5 dpi	33.41	28.50	4.27	98.81	96.22	85.31
Pt 6 dpi	32.89	28.23	4.23	98.77	96.49	85.84
Pt CK	32.00	27.15	4.07	98.87	96.31	84.85
Pm 1dpi	35.76	30.19	4.53	98.80	95.99	84.43
Pm 2 dpi	34.98	30.04	4.50	98.68	95.89	85.89
Pm 3 dpi	35.09	29.95	4.49	98.79	95.77	85.36
Pm 4 dpi	35.55	30.28	4.54	98.83	96.02	85.19
Pm 5 dpi	34.68	29.86	4.48	98.88	96.21	86.11
Pm 6 dpi	35.77	30.97	4.64	98.97	96.19	86.58
Pm CK	35.80	30.22	4.53	98.78	96.16	84.41

Table S2. Number of genes in each module.

Module	Gene number
black	56
blue	1349
brown	427
green	254
grey	114
red	108
turquoise	9275
yellow	278

Table S3. GO enrichment result for genes in the turquoise module (top 20 terms for each class).

ID	Count	Class	Description
GO:0008150	2056	Biological Process	biological process
GO:0008152	1551	Biological Process	metabolic process
GO:0071704	1107	Biological Process	organic substance metabolic process
GO:0009987	1093	Biological Process	cellular process
GO:0044238	882	Biological Process	primary metabolic process
GO:0044237	871	Biological Process	cellular metabolic process
GO:0043170	720	Biological Process	macromolecule metabolic process
GO:0044699	649	Biological Process	single-organism process
GO:0044763	512	Biological Process	single-organism cellular process
GO:0044260	452	Biological Process	cellular macromolecule metabolic process
GO:0044710	437	Biological Process	single-organism metabolic process
GO:0006807	413	Biological Process	nitrogen compound metabolic process
GO:0019538	352	Biological Process	protein metabolic process
GO:0044281	346	Biological Process	small molecule metabolic process
GO:0034641	334	Biological Process	cellular nitrogen compound metabolic process
GO:0010467	326	Biological Process	gene expression
GO:1901360	319	Biological Process	organic cyclic compound metabolic process
GO:0006725	310	Biological Process	cellular aromatic compound metabolic process
GO:0051179	307	Biological Process	localization
GO:0046483	304	Biological Process	heterocycle metabolic process
GO:0005575	1473	Cellular Component	cellular component
GO:0005623	1196	Cellular Component	cell
GO:0044464	1196	Cellular Component	cell part
GO:0005622	1009	Cellular Component	intracellular
GO:0044424	993	Cellular Component	intracellular part
GO:0043226	699	Cellular Component	organelle
GO:0043229	693	Cellular Component	intracellular organelle
GO:0043227	598	Cellular Component	membrane-bounded organelle
GO:0043231	586	Cellular Component	intracellular membrane-bounded organelle
GO:0016020	363	Cellular Component	membrane
GO:0032991	321	Cellular Component	macromolecular complex
GO:0005737	284	Cellular Component	cytoplasm
GO:0044444	281	Cellular Component	cytoplasmic part
GO:0044425	205	Cellular Component	membrane part
GO:0030529	170	Cellular Component	intracellular ribonucleoprotein complex
GO:1990904	170	Cellular Component	ribonucleoprotein complex
GO:0031224	165	Cellular Component	intrinsic component of membrane
GO:0009536	163	Cellular Component	plastid
GO:0044422	160	Cellular Component	organelle part
GO:0044446	150	Cellular Component	intracellular organelle part
GO:0003674	2655	Molecular Function	molecular function
GO:0003824	1752	Molecular Function	catalytic activity

GO:0005488	1523	Molecular Function	binding
GO:0097159	761	Molecular Function	organic cyclic compound binding
GO:1901363	761	Molecular Function	heterocyclic compound binding
GO:0016740	542	Molecular Function	transferase activity
GO:0016787	525	Molecular Function	hydrolase activity
GO:0036094	514	Molecular Function	small molecule binding
GO:0000166	501	Molecular Function	nucleotide binding
GO:1901265	501	Molecular Function	nucleoside phosphate binding
GO:0043167	459	Molecular Function	ion binding
GO:0043169	432	Molecular Function	cation binding
GO:0097367	381	Molecular Function	carbohydrate derivative binding
GO:0017076	380	Molecular Function	purine nucleotide binding
GO:0032553	378	Molecular Function	ribonucleotide binding
GO:0032555	378	Molecular Function	purine ribonucleotide binding
GO:0046872	366	Molecular Function	metal ion binding
GO:0030554	299	Molecular Function	adenyl nucleotide binding
GO:0032559	297	Molecular Function	adenyl ribonucleotide binding
GO:0003676	281	Molecular Function	nucleic acid binding

Table S4. GO enrichment result for genes in the yellow module (top 20 terms for each class).

ID	Count	Class	Description
GO:0008150	79	Biological Process	biological process
GO:0008152	69	Biological Process	metabolic process
GO:0009987	42	Biological Process	cellular process
GO:0044237	35	Biological Process	cellular metabolic process
GO:0071704	34	Biological Process	organic substance metabolic process
GO:0044238	30	Biological Process	primary metabolic process
GO:0044699	28	Biological Process	single-organism process
GO:0044710	23	Biological Process	single-organism metabolic process
GO:0044763	22	Biological Process	single-organism cellular process
GO:0044281	17	Biological Process	small molecule metabolic process
GO:0006807	13	Biological Process	nitrogen compound metabolic process
GO:0043170	12	Biological Process	macromolecule metabolic process
GO:0006082	10	Biological Process	organic acid metabolic process
GO:0006725	10	Biological Process	cellular aromatic compound metabolic process
GO:0009058	10	Biological Process	biosynthetic process
GO:0019538	10	Biological Process	protein metabolic process
GO:0019752	10	Biological Process	carboxylic acid metabolic process
GO:0043436	10	Biological Process	oxoacid metabolic process
GO:0044260	10	Biological Process	cellular macromolecule metabolic process
GO:1901360	9	Biological Process	organic cyclic compound metabolic process
GO:0005575	35	Cellular Component	cellular component
GO:0005623	30	Cellular Component	cell
GO:0044464	30	Cellular Component	cell part
GO:0005622	22	Cellular Component	intracellular
GO:0044424	22	Cellular Component	intracellular part
GO:0043226	15	Cellular Component	organelle
GO:0043229	15	Cellular Component	intracellular organelle
GO:0043227	13	Cellular Component	membrane-bounded organelle
GO:0043231	13	Cellular Component	intracellular membrane-bounded organelle
GO:0005737	8	Cellular Component	cytoplasm
GO:0044444	8	Cellular Component	cytoplasmic part
GO:0016020	6	Cellular Component	membrane
GO:0032991	5	Cellular Component	macromolecular complex
GO:0043234	4	Cellular Component	protein complex
GO:0009536	3	Cellular Component	plastid
GO:0031410	3	Cellular Component	cytoplasmic vesicle
GO:0031982	3	Cellular Component	vesicle
GO:0044425	3	Cellular Component	membrane part
GO:0031224	2	Cellular Component	intrinsic component of membrane
GO:0042579	2	Cellular Component	microbody
GO:0003674	109	Molecular Function	molecular function
GO:0003824	89	Molecular Function	catalytic activity

GO:0005488	55	Molecular Function	binding
GO:0016740	30	Molecular Function	transferase activity
GO:0016491	21	Molecular Function	oxidoreductase activity
GO:0043167	20	Molecular Function	ion binding
GO:0097159	20	Molecular Function	organic cyclic compound binding
GO:1901363	20	Molecular Function	heterocyclic compound binding
GO:0043169	19	Molecular Function	cation binding
GO:0046872	18	Molecular Function	metal ion binding
GO:0036094	17	Molecular Function	small molecule binding
GO:0000166	16	Molecular Function	nucleotide binding
GO:1901265	16	Molecular Function	nucleoside phosphate binding
GO:0016787	14	Molecular Function	hydrolase activity
GO:0017076	12	Molecular Function	purine nucleotide binding
GO:0032553	12	Molecular Function	ribonucleotide binding
GO:0032555	12	Molecular Function	purine ribonucleotide binding
GO:0097367	12	Molecular Function	carbohydrate derivative binding
GO:0046914	11	Molecular Function	transition metal ion binding
GO:0030554	10	Molecular Function	adenyl nucleotide binding

Table S5. KEGG enrichment result for genes in the turquoise module (top 20 pathways).

KEGG A class	KEGG B class	Pathway	Count (2731 in total)	Pathway ID
Metabolism	Global and overview maps	Metabolic pathways	1184	ko01100
Metabolism	Global and overview maps	Biosynthesis of secondary metabolites	691	ko01110
Genetic Information Processing	Translation	Ribosome	162	ko03010
Genetic Information Processing	Transcription	Spliceosome	144	ko03040
Environmental Information Processing	Signal transduction	Plant hormone signal transduction	135	ko04075
Genetic Information Processing	Folding, sorting and degradation	Protein processing in endoplasmic reticulum	127	ko04141
Metabolism	Global and overview maps	Carbon metabolism	119	ko01200
Organismal Systems	Environmental adaptation	Plant-pathogen interaction	114	ko04626
Genetic Information Processing	Translation	RNA transport	111	ko03013
Environmental Information Processing	Signal transduction	MAPK signaling pathway - plant	106	ko04016
Metabolism	Carbohydrate metabolism	Starch and sucrose metabolism	93	ko00500
Metabolism	Biosynthesis of other secondary metabolites	Phenylpropanoid biosynthesis	93	ko00940
Metabolism	Global and overview maps	Biosynthesis of amino acids	92	ko01230
Cellular Processes	Transport and catabolism	Endocytosis	90	ko04144
Genetic Information Processing	Translation	mRNA surveillance pathway	79	ko03015
Genetic Information Processing	Folding, sorting and degradation	Ubiquitin mediated proteolysis	66	ko04120
Metabolism	Biosynthesis of other secondary metabolites	Flavonoid biosynthesis	65	ko00941
Metabolism	Carbohydrate metabolism	Amino sugar and nucleotide sugar metabolism	64	ko00520
Genetic Information Processing	Folding, sorting and degradation	RNA degradation	63	ko03018
Genetic Information Processing	Translation	Ribosome biogenesis in eukaryotes	60	ko03008

Table S6. KEGG enrichment result for genes in the yellow module (top 20 pathways).

KEGG A class	KEGG B class	Pathway	Count (120 in total)	Pathway ID
Metabolism	Global and overview maps	Metabolic pathways	78	ko01100
Metabolism	Global and overview maps	Biosynthesis of secondary metabolites	68	ko01110
Metabolism	Biosynthesis of other secondary metabolites	Flavonoid biosynthesis	30	ko00941
Metabolism	Global and overview maps	Biosynthesis of amino acids	12	ko01230
Metabolism	Biosynthesis of other secondary metabolites	Phenylpropanoid biosynthesis	8	ko00940
Metabolism	Global and overview maps	Carbon metabolism	8	ko01200
Environmental Information Processing	Signal transduction	Plant hormone signal transduction	8	ko04075
Cellular Processes	Transport and catabolism	Endocytosis	6	ko04144
Metabolism	Carbohydrate metabolism	Glycolysis / Gluconeogenesis	5	ko00010
Metabolism	Biosynthesis of other secondary metabolites	Flavone and flavonol biosynthesis	5	ko00944
Environmental Information Processing	Membrane transport	ABC transporters	5	ko02010
Environmental Information Processing	Signal transduction	MAPK signaling pathway - plant	5	ko04016
Organismal Systems	Environmental adaptation	Circadian rhythm - plant	5	ko04712
Metabolism	Amino acid metabolism	Alanine, aspartate and glutamate metabolism	4	ko00250
Genetic Information Processing	Transcription	Spliceosome	4	ko03040
Metabolism	Carbohydrate metabolism	Fructose and mannose metabolism	3	ko00051
Metabolism	Lipid metabolism	Fatty acid degradation	3	ko00071
Metabolism	Lipid metabolism	Cutin, suberine and wax biosynthesis	3	ko00073
Metabolism	Amino acid metabolism	Cysteine and methionine metabolism	3	ko00270
Metabolism	Amino acid metabolism	Phenylalanine, tyrosine and tryptophan biosynthesis	3	ko00400

Table S7. Genes with the top 30 highest IC values in the turquoise module.

No.	Gene annotation	IC	Homologous protein GenBank ID	Protein name	Blast nr *p* value
1	outer membrane family protein	1734.57	XP_008803105.1	PREDICTED: protein TOC75-3, chloroplastic-like [*Phoenix dactylifera*]	6.78×10^{-159}
2	Dof11	1715.35	CAP04930.1	dof transcription factor [*Pinus pinaster*]	8.43×10^{-128}
3	pentatricopeptide repeat-containing protein chloroplastic	1715.25	XP_010275281.1	PREDICTED: pentatricopeptide repeat-containing protein At5g46580, chloroplastic [*Nelumbo nucifera*]	0
4	KH domain-containing protein	1700.30	ABK24418.1	unknown [*Picea sitchensis*]	2.07×10^{-51}
5	Lysyl-tRNA synthetase	1699.37	XP_010911688.1	PREDICTED: lysine--tRNA ligase-like isoform X2 [*Elaeis guineensis*]	0
6	transport inhibitor response 1-like protein	1690.19	KHG29069.1	hypothetical protein F383_04554 [*Gossypium arboreum*]	0
7	heterodimeric geranylgeranyl pyrophosphate synthase small subunit, chloroplastic-like	1681.73	ACN40123.1	unknown [*Picea sitchensis*]	1.36×10^{-173}
8	uninformative	1680.43	AEW09324.1	hypothetical protein UMN_4541_01, partial [*Pinus radiata*]	6.74×10^{-71}
9	DCD domain protein	1679.25	ABK22365.1	unknown [*Picea sitchensis*]	5.34×10^{-127}
10	ruBisCO large subunit-binding protein subunit beta, chloroplastic	1677.41	ABK24483.1	unknown [*Picea sitchensis*]	0
11	DNA-directed RNA polymerase I subunit RPA2-like isoform X1	1676.70	XP_010659083.1	PREDICTED: DNA-directed RNA polymerase I subunit RPA2 isoform X2 [*Vitis vinifera*]	0
12	probable E3 ubiquitin-protein ligase ARI11	1671.09	ADE76274.1	unknown [*Picea sitchensis*]	0
13	uninformative	1665.32	ABR18130.1	unknown [*Picea sitchensis*]	1.02×10^{-110}
14	not annotated	1665.19	ADE76372.1	unknown [*Picea sitchensis*]	0
15	ruBisCO large subunit-binding	1661.12	ABR18037.1	unknown [*Picea sitchensis*]	1.01×10^{-114}

	protein subunit alpha, chloroplastic-like				
16	scarecrow-like protein 6-like	1659.92	AJP06299.1	HAM1 [*Pinus tabuliformis*]	0
17	not annotated	1653.47	XP_010270433.1	PREDICTED: ATP-dependent zinc metalloprotease FtsH [*Nelumbo nucifera*]	0
18	not annotated	1651.52	ACN40698.1	unknown [*Picea sitchensis*]	1.11×10^{-75}
19	long chain acyl-CoA synthetase 9, chloroplastic	1650.71	XP_010912477.1	PREDICTED: long chain acyl-CoA synthetase 9, chloroplastic-like [*Elaeis guineensis*]	0
20	extensin-like protein	1647.48	ADM76665.1	extensin-like protein [*Picea sitchensis*]	8.90×10^{-60}
21	auxin response factor 17-like	1637.60	CBA12003.1	putative auxin response factor 6/8 [*Ginkgo biloba*]	7.44×10^{-59}
22	nucleolin	1632.70	XP_006838503.1	PREDICTED: nucleolin [*Amborella trichopoda*]	6.96×10^{-128}
23	ubiquitin interaction motif-containing protein	1629.22	XP_006383620.1	hypothetical protein POPTR_0005s21350g [*Populus trichocarpa*]	0
24	RNA binding protein	1626.72	ADE76786.1	unknown [*Picea sitchensis*]	5.02×10^{-45}
25	Patellin-6	1623.89	XP_006828153.1	PREDICTED: patellin-6 [*Amborella trichopoda*]	9.14×10^{-41}
26	catalase 2	1618.60	ABK24449.1	unknown [*Picea sitchensis*]	0
27	N-alpha-acetyltransferase 15, NatA auxiliary subunit-like isoform X1	1617.71	XP_008792977.1	PREDICTED: N-alpha-acetyltransferase 16, NatA auxiliary subunit-like [*Phoenix dactylifera*]	0
28	not annotated	1614.17	ABK24027.1	unknown [*Picea sitchensis*]	1.14×10^{-155}
29	homeobox transcription factor Hox7	1613.32	XP_008795938.1	PREDICTED: uncharacterized protein LOC103711537 [*Phoenix dactylifera*]	4.42×10^{-75}
30	glycine-rich RNA-binding protein 3	1613.31	XP_009787255.1	PREDICTED: glycine-rich RNA-binding protein 3, mitochondrial-like [*Nicotiana sylvestris*]	7.19×10^{-24}

Table S8. Genes with the top 30 highest IC values in the yellow module.

No.	Gene annotation	IC	Homologous protein GenBank ID	Protein name	Blast nr *p* value
1	(+)-pulegone reductase-like, partial	56.68	ABR17262.1	unknown [*Picea sitchensis*]	8.48×10^{-113}
2	chalcone-flavonone isomerase 2	56.14	ADE77594.1	unknown [*Picea sitchensis*]	0
3	chalcone synthase 2	55.53	AEN84256.1	chalcone synthase, partial [*Picea sitchensis*]	0
4	digalactosyldiacylglycerol synthase 1, chloroplastic	53.75	XP_002264659.1	PREDICTED: digalactosyldiacylglycerol synthase 1, chloroplastic [*Vitis vinifera*]	0
5	chalcone synthase 2	52.19	CAA05214.1	chalcone synthase-like protein [*Pinus strobus*]	0
6	NADPH oxidoreductase	51.84	CAI56321.1	TPA: leucoanthocyanidin reductase [*Pinus taeda*]	0
7	chalcone synthase	51.79	AEN84256.1	chalcone synthase, partial [*Picea sitchensis*]	5.24×10^{-129}
8	receptor-like protein kinase HSL1-like	51.50	XP_012092411.1	PREDICTED: receptor-like protein kinase HSL1 [*Jatropha curcas*]	0
9	1-aminocyclopropane-1-carboxylate synthase	51.49	ABM60748.1	ACC synthase [*Picea engelmannii* × *Picea glauca*]	0
10	pleiotropic drug resistance protein 1-like	51.28	XP_010249930.1	PREDICTED: pleiotropic drug resistance protein 1-like [*Nelumbo nucifera*]	2.45×10^{-134}
11	chorismate mutase	51.10	XP_006857227.2	PREDICTED: chorismate mutase 1, chloroplastic [*Amborella trichopoda*]	2.02×10^{-85}
12	Os09g0332600	50.91	KVI05977.1	hypothetical protein Ccrd_015657 [*Cynara cardunculus* var. *scolymus*]	0
13	cinnamoyl-CoA reductase, putative	50.89	AFC38436.1	cinnamoyl-CoA reductase [*Pinus radiata*]	4.04×10^{-161}
14	leucoanthocyanidin dioxygenase family protein	49.38	AHW42460.1	GA2ox10 [*Pinus tabuliformis*]	3.20×10^{-177}
15	chalcone synthase	48.20	AGU43679.1	pinocembrin chalcone synthase [*Pinus massoniana*]	0
16	LOW QUALITY PROTEIN: leucoanthocyanidin reductase-like	47.75	AHB89627.1	leucoanthocyanidin reductase 1 [*Picea abies*]	2.07×10^{-180}

17	phospholipid-transporting ATPase 1	47.63	XP_002271241.2	PREDICTED: phospholipid-transporting ATPase 1 [*Vitis vinifera*]	0
18	AMP dependent CoA ligase, putative	47.62	AGX45528.1	4-coumarate:CoA ligase [*Pinus taeda*]	0
19	chalcone synthase 1-like	47.59	ABK24577.1	unknown [*Picea sitchensis*]	0
20	dihydrolipoyllysine-residue succinyltransferase component of 2-oxoglutarate dehydrogenase complex 1, mitochondrial	46.88	ADE77401.1	unknown [*Picea sitchensis*]	0
21	oxalate-CoA ligase-like	46.50	ABR16382.1	unknown [*Picea sitchensis*]	0
22	kinase	46.33	XP_006856200.1	PREDICTED: wall-associated receptor kinase-like 14 [*Amborella trichopoda*]	4.05×10^{-163}
23	not annotated	46.23	ACN40312.1	unknown [*Picea sitchensis*]	0
24	3-ketoacyl-CoA thiolase B	45.45	EMS60447.1	3-ketoacyl-CoA thiolase 2, peroxisomal [*Triticum urartu*]	1.67×10^{-93}
25	ATP-binding cassette transporter	45.27	XP_010273226.1	PREDICTED: pleiotropic drug resistance protein 1-like [*Nelumbo nucifera*]	3.94×10^{-118}
26	uninformative	45.26	ABK27055.1	unknown [*Picea sitchensis*]	1.26×10^{-29}
27	flavonoid 3'-monooxygenase	45.11	ABR16821.1	unknown [*Picea sitchensis*]	9.19×10^{-146}
28	probable voltage-gated potassium channel subunit beta-like	45.04	ABK21055.1	unknown [*Picea sitchensis*]	0
29	trans-cinnamate 4-monooxygenase-like	45.03	AAD23378.1	trans-cinnamate 4-hydroxylase [*Pinus taeda*]	0
30	leucoanthocyanidin dioxygenase family protein	44.96	AHW42460.1	GA2ox10 [*Pinus tabuliformis*]	2.34×10^{-166}

Table S9. KEGG enrichment result for genes with the top 30 highest IC values in the turquoise module.

KEGG A class	KEGG B class	Pathway	Count (10 in total)	Pathway ID
Environmental Information Processing	Signal transduction	Plant hormone signal transduction	3	ko04075
Metabolism	Global and overview maps	Metabolic pathways	2	ko01100
Genetic Information Processing	Folding, sorting and degradation	RNA degradation	2	ko03018
Cellular Processes	Transport and catabolism	Peroxisome	2	ko04146
Metabolism	Lipid metabolism	Fatty acid biosynthesis	1	ko00061
Metabolism	Lipid metabolism	Fatty acid degradation	1	ko00071
Metabolism	Amino acid metabolism	Tryptophan metabolism	1	ko00380
Metabolism	Carbohydrate metabolism	Glyoxylate and dicarboxylate metabolism	1	ko00630
Genetic Information Processing	Translation	Aminoacyl-tRNA biosynthesis	1	ko00970
Metabolism	Global and overview maps	Biosynthesis of secondary metabolites	1	ko01110
Metabolism	Global and overview maps	Carbon metabolism	1	ko01200
Metabolism	Global and overview maps	Fatty acid metabolism	1	ko01212
Genetic Information Processing	Transcription	Spliceosome	1	ko03040
Environmental Information Processing	Signal transduction	MAPK signaling pathway - plant	1	ko04016
Organismal Systems	Environmental adaptation	Circadian rhythm - plant	1	ko04712

Table S10. Genes with the top 30 highest negative value of GS values for nematode population in the turquoise module.

No.	Gene annotation	GS	GS *p* value	Homologous protein GenBank ID	Blast nr *p* value	Protein name
1	starch synthase 1, chloroplastic/amyloplastic	-0.8941	3.01638×10^{-06}	XP_006852610.1	2.19×10^{-133}	PREDICTED: soluble starch synthase 1, chloroplastic/amyloplastic [*Amborella trichopoda*]
2	serine/threonine-protein phosphatase 4 regulatory subunit 3-like	-0.8851	5.19811×10^{-06}	XP_008808656.1	2.26×10^{-68}	PREDICTED: serine/threonine-protein phosphatase 4 regulatory subunit 3B-like isoform X1 [*Phoenix dactylifera*]
3	nuclear pore complex protein NUP88	-0.8724	1.04655×10^{-05}	XP_011623988.1	0	PREDICTED: nuclear pore complex protein NUP88 [*Amborella trichopoda*]
4	AMP deaminase, putative	-0.8647	1.54239×10^{-05}	XP_015582669.1	7.28×10^{-88}	PREDICTED: AMP deaminase [*Ricinus communis*]
5	homeobox-leucine zipper protein ATHB-13	-0.8634	1.64552×10^{-05}	ABK24498.1	3.39×10^{-128}	unknown [*Picea sitchensis*]
6	uninformative	-0.8629	1.68884×10^{-05}	ERN01515.1	0	hypothetical protein AMTR_s00002p00270610 [*Amborella trichopoda*]
7	chaperone protein ClpB3, mitochondrial-like isoform X1	-0.8528	2.69559×10^{-05}	XP_010665999.1	0	PREDICTED: chaperone protein ClpB4, mitochondrial [*Beta vulgaris* subsp. *vulgaris*]
8	awaiting functional assignment	-0.8507	2.9658×10^{-05}	XP_006577150.1	1.36×10^{-132}	PREDICTED: uncharacterized protein C57A7.06-like isoform X1 [*Glycine max*]
9	amidophosphoribosyltransferase 2, chloroplastic-like	-0.8488	3.21936×10^{-05}	XP_010247381.1	0	PREDICTED: amidophosphoribosyltransferase, chloroplastic-like [*Nelumbo nucifera*]
10	uncharacterized protein isoform 1	-0.8484	3.27887×10^{-05}	XP_010263219.1	4.20×10^{-53}	PREDICTED: uncharacterized protein LOC104601549 [*Nelumbo nucifera*]
11	uninformative	-0.8473	3.42711×10^{-05}	ABK25240.1	0	unknown [*Picea sitchensis*]
12	not annotated	-0.8459	3.63909×10^{-05}	ADE75793.1	3.00×10^{-71}	unknown [*Picea sitchensis*]
13	uninformative	-0.8459	3.65153×10^{-05}	XP_001766925.1	3.68×10^{-98}	predicted protein [*Physcomitrella patens*]
14	not annotated	-0.8424	4.21612×10^{-05}	XP_011620903.1	4.63×10^{-66}	PREDICTED: uncharacterized protein LOC18427592 isoform X2 [*Amborella trichopoda*]
15	not annotated	-0.8414	4.39415×10^{-05}	XP_006853427.1	7.03×10^{-115}	PREDICTED: uncharacterized protein At4g19900 isoform X1 [*Amborella trichopoda*]

16	putative pentatricopeptide repeat-containing protein At5g06400, mitochondrial	-0.8405	4.57032×10^{-05}	XP_010938939.1	0	PREDICTED: putative pentatricopeptide repeat-containing protein At5g06400, mitochondrial [*Elaeis guineensis*]
17	methyl-CpG-binding domain-containing protein 5-like	-0.8398	4.70182×10^{-05}	XP_011624098.1	5.71×10^{-23}	PREDICTED: methyl-CpG-binding domain-containing protein 5 isoform X3 [*Amborella trichopoda*]
18	GCN5-like N-acetyltransferase	-0.8395	4.75821×10^{-05}	ABK22519.1	7.28×10^{-111}	unknown [*Picea sitchensis*]
19	not annotated	-0.8391	4.83334×10^{-05}	XP_006376149.1	2.06×10^{-67}	hypothetical protein POPTR_0013s10250g [*Populus trichocarpa*]
20	pentatricopeptide repeat-containing protein At4g20740-like	-0.8381	5.02879×10^{-05}	XP_010255813.1	0	PREDICTED: pentatricopeptide repeat-containing protein At4g20740 [*Nelumbo nucifera*]
21	not annotated	-0.8380	5.05526×10^{-05}	XP_010259232.1	5.38×10^{-81}	PREDICTED: DNA replication complex GINS protein SLD5 [*Nelumbo nucifera*]
22	RNA recognition motif-containing protein	-0.8379	5.07924×10^{-05}	ADE77701.1	5.27×10^{-107}	unknown [*Picea sitchensis*]
23	not annotated	-0.8378	5.10092×10^{-05}	ABR17863.1	4.48×10^{-160}	unknown [*Picea sitchensis*]
24	glucan synthase-like 3	-0.8359	5.51103×10^{-05}	XP_009608250.1	1.92×10^{-52}	PREDICTED: callose synthase 3-like [*Nicotiana tomentosiformis*]
25	uninformative	-0.8345	5.80868×10^{-05}	XP_010272245.1	0	PREDICTED: protein furry homolog-like [*Nelumbo nucifera*]
26	uninformative	-0.8330	6.1702×10^{-05}	ADE77306.1	1.03×10^{-55}	unknown [*Picea sitchensis*]
27	dihydropyrimidinase-like	-0.8326	6.26223×10^{-05}	ABR16534.1	1.10×10^{-115}	unknown [*Picea sitchensis*]
28	uninformative	-0.8322	6.35978×10^{-05}	XP_002967506.1	1.33×10^{-78}	hypothetical protein SELMODRAFT_408574 [*Selaginella moellendorffii*]
29	not annotated	-0.8321	6.38739×10^{-05}	ABR16124.1	0	unknown [*Picea sitchensis*]
30	tetraspanin family protein	-0.8313	6.58096×10^{-05}	ADE76479.1	6.45×10^{-89}	unknown [*Picea sitchensis*]

Table S11. KEGG enrichment result for genes with the top 30 highest negative GS values in the turquoise module.

KEGG A class	KEGG B class	Pathway	Count (7 in total)	Pathway ID
Metabolism	Global and overview maps	Metabolic pathways	3	ko01100
Metabolism	Global and overview maps	Biosynthesis of secondary metabolites	2	ko01110
Metabolism	Nucleotide metabolism	Purine metabolism	1	ko00230
Metabolism	Nucleotide metabolism	Pyrimidine metabolism	1	ko00240
Metabolism	Amino acid metabolism	Alanine, aspartate and glutamate metabolism	1	ko00250
Metabolism	Metabolism of other amino acids	beta-Alanine metabolism	1	ko00410
Metabolism	Carbohydrate metabolism	Starch and sucrose metabolism	1	ko00500
Metabolism	Metabolism of cofactors and vitamins	Pantothenate and CoA biosynthesis	1	ko00770
Genetic Information Processing	Translation	Ribosome biogenesis in eukaryotes	1	ko03008
Genetic Information Processing	Translation	RNA transport	1	ko03013
Genetic Information Processing	Folding, sorting and degradation	Protein export	1	ko03060
Cellular Processes	Transport and catabolism	Peroxisome	1	ko04146

Table S12. KEGG enrichment result for genes with the top 30 highest IC values in the yellow module.

KEGG A class	KEGG B class	Pathway	Count (18 in total)	Pathway ID
Metabolism	Global and overview maps	Metabolic pathways	15	ko01100
Metabolism	Global and overview maps	Biosynthesis of secondary metabolites	15	ko01110
Metabolism	Biosynthesis of other secondary metabolites	Flavonoid biosynthesis	9	ko00941
Organismal Systems	Environmental adaptation	Circadian rhythm - plant	3	ko04712
Metabolism	Biosynthesis of other secondary metabolites	Phenylpropanoid biosynthesis	2	ko00940
Metabolism	Global and overview maps	Carbon metabolism	2	ko01200
Metabolism	Carbohydrate metabolism	Glycolysis / Gluconeogenesis	1	ko00010
Metabolism	Carbohydrate metabolism	Citrate cycle (TCA cycle)	1	ko00020
Metabolism	Metabolism of cofactors and vitamins	Ubiquinone and other terpenoid-quinone biosynthesis	1	ko00130
Metabolism	Amino acid metabolism	Cysteine and methionine metabolism	1	ko00270
Metabolism	Amino acid metabolism	Lysine degradation	1	ko00310
Metabolism	Amino acid metabolism	Tryptophan metabolism	1	ko00380
Metabolism	Amino acid metabolism	Phenylalanine, tyrosine and tryptophan biosynthesis	1	ko00400
Metabolism	Lipid metabolism	Glycerolipid metabolism	1	ko00561
Metabolism	Carbohydrate metabolism	Pyruvate metabolism	1	ko00620
Metabolism	Carbohydrate metabolism	Glyoxylate and dicarboxylate metabolism	1	ko00630
Metabolism	Carbohydrate metabolism	Propanoate metabolism	1	ko00640
Metabolism	Metabolism of cofactors and vitamins	Vitamin B6 metabolism	1	ko00750
Metabolism	Biosynthesis of other secondary metabolites	Flavone and flavonol biosynthesis	1	ko00944
Metabolism	Global and overview maps	Biosynthesis of amino acids	1	ko01230
Environmental Information Processing	Membrane transport	ABC transporters	1	ko02010
Environmental Information Processing	Signal transduction	MAPK signaling pathway - plant	1	ko04016

Table S13. Genes with the top 30 highest GS values for nematode population in the yellow module.

No.	Gene annotation	GS	GS<br>*p* value	Homologous protein GenBank ID	Blast nr<br>*p* value	Protein name
1	serine carboxypeptidase-like 20-like	0.8481	3.31576×10^{-05}	ABR16352.1	2.10×10^{-136}	unknown [*Picea sitchensis*]
2	DNA binding protein	0.8375	5.16089×10^{-05}	XP_008782582.1	1.20×10^{-29}	PREDICTED: putative transcription factor bHLH041 [*Phoenix dactylifera*]
3	serine carboxypeptidase-like 20-like	0.8366	5.34412×10^{-05}	ABR16352.1	0	unknown [*Picea sitchensis*]
4	3-ketoacyl-CoA thiolase 2	0.8271	7.7402×10^{-05}	ADE77908.1	0	unknown [*Picea sitchensis*]
5	flavonoid 3'-monooxygenase-like	0.8260	8.04993×10^{-05}	ABR16821.1	2.27×10^{-136}	unknown [*Picea sitchensis*]
6	flavonoid 3'-monooxygenase	0.8229	9.05631×10-05	ABR16821.1	9.19×10^{-146}	unknown [*Picea sitchensis*]
7	serine carboxypeptidase-like 3	0.8101	0.000142025	XP_010936152.1	3.08×10^{-67}	PREDICTED: serine carboxypeptidase-like 18 isoform X2 [*Elaeis guineensis*]
8	pleiotropic drug resistance protein 1-like	0.8078	0.000153718	XP_010273226.1	3.32×10^{-143}	PREDICTED: pleiotropic drug resistance protein 1-like [*Nelumbo nucifera*]
9	UPF0468 protein CG5343-like	0.8005	0.00019535	ABK25880.1	6.61×10^{-39}	unknown [*Picea sitchensis*]
10	alpha-1,6-mannosyl-glycoprotein 2-beta-N-acetylglucosaminyltransferase-like	0.7960	0.000225439	XP_010906223.1	3.13×10^{-160}	PREDICTED: alpha-1,6-mannosyl-glycoprotein 2-beta-N-acetylglucosaminyltransferase [*Elaeis guineensis*]
11	gd2b, putative	0.7959	0.000226119	ABR18114.1	0	unknown [*Picea sitchensis*]
12	14-3-3-like protein-like	0.7916	0.00025844	ABK21293.1	1.33×10^{-143}	unknown [*Picea sitchensis*]
13	digalactosyldiacylglycerol synthase 1, chloroplastic	0.7881	0.000287973	XP_002264659.1	0	PREDICTED: digalactosyldiacylglycerol synthase 1, chloroplastic [*Vitis vinifera*]
14	probable mannitol dehydrogenase-like	0.7782	0.000385345	ABK26008.1	2.27×10^{-153}	unknown [*Picea sitchensis*]
15	FAD-dependent urate hydroxylase-like	0.7773	0.000394403	XP_006838554.1	5.61×10^{-145}	PREDICTED: zeaxanthin epoxidase, chloroplastic [*Amborella trichopoda*]

16	not annotated	0.7762	0.000407237	ACN40312.1	0	unknown [*Picea sitchensis*]
17	uninformative	0.7734	0.000441315	B8LPG7.1	2.77×10^{-128}	RecName: Full=Anamorsin homolog 2; AltName: Full=Fe-S cluster assembly protein DRE2 homolog 2
18	unknown	0.7684	0.000506061	ABR18272.1	2.43×10^{-53}	unknown [*Picea sitchensis*]
19	protein SRG1-like	0.7639	0.000572338	AAA85365.1	7.17×10^{-115}	ethylene-forming enzyme [*Picea glauca*]
20	uninformative	0.7608	0.000621668	XP_010939602.1	0	PREDICTED: uncharacterized protein LOC105058381 isoform X2 [*Elaeis guineensis*]
21	NADPH oxidoreductase, putative	0.7582	0.000664766	CAI56321.1	0	TPA: leucoanthocyanidin reductase [*Pinus taeda*]
22	major facilitator family protein	0.7570	0.000685331	ABR18255.1	0	unknown [*Picea sitchensis*]
23	Transcription factor AtMYC2, putative	0.7511	0.000797278	ABR16623.1	0	unknown [*Picea sitchensis*]
24	proline-rich receptor-like protein kinase PERK13-like	0.7509	0.000802253	XP_011625141.1	0	PREDICTED: chitin elicitor receptor kinase 1 [*Amborella trichopoda*]
25	not annotated	0.7482	0.000857794	ABK21817.1	3.60×10^{-69}	unknown [*Picea sitchensis*]
26	1,4-dihydroxy-2-naphthoyl-CoA thioesterase 1	0.7471	0.000881101	ABK24103.1	1.66×10^{-81}	unknown [*Picea sitchensis*]
27	MATE efflux family protein 6 isoform X1	0.7466	0.00089286	XP_006843973.1	3.35×10^{-150}	PREDICTED: MATE efflux family protein 5 isoform X1 [*Amborella trichopoda*]
28	(+)-pulegone reductase-like, partial	0.7446	0.000936378	ABR17262.1	8.48×10^{-113}	unknown [*Picea sitchensis*]
29	chalcone synthase 1 isoform X1	0.7445	0.000938956	AGY80771.1	0	chalcone synthase [*Pinus radiata*]
30	Chalcone synthase	0.7420	0.000999184	AGU43679.1	0	pinocembrin chalcone synthase [*Pinus massoniana*]

Table S14. KEGG enrichment result for genes with the top 30 highest GS values in the yellow module.

KEGG A class	KEGG B class	Pathway	Count (15 in total)	Pathway ID
Metabolism	Global and overview maps	Metabolic pathways	11	ko01100
Metabolism	Global and overview maps	Biosynthesis of secondary metabolites	9	ko01110
Metabolism	Biosynthesis of other secondary metabolites	Flavonoid biosynthesis	6	ko00941
Metabolism	Biosynthesis of other secondary metabolites	Flavone and flavonol biosynthesis	2	ko00944
Environmental Information Processing	Signal transduction	Plant hormone signal transduction	2	ko04075
Organismal Systems	Environmental adaptation	Circadian rhythm - plant	2	ko04712
Metabolism	Carbohydrate metabolism	Glycolysis / Gluconeogenesis	1	ko00010
Metabolism	Lipid metabolism	Fatty acid degradation	1	ko00071
Metabolism	Amino acid metabolism	Valine, leucine and isoleucine degradation	1	ko00280
Metabolism	Glycan biosynthesis and metabolism	N-Glycan biosynthesis	1	ko00510
Metabolism	Glycan biosynthesis and metabolism	Various types of N-glycan biosynthesis	1	ko00513
Metabolism	Lipid metabolism	Glycerolipid metabolism	1	ko00561
Metabolism	Lipid metabolism	alpha-Linolenic acid metabolism	1	ko00592
Metabolism	Carbohydrate metabolism	Pyruvate metabolism	1	ko00620
Metabolism	Carbohydrate metabolism	Glyoxylate and dicarboxylate metabolism	1	ko00630
Metabolism	Carbohydrate metabolism	Propanoate metabolism	1	ko00640
Metabolism	Metabolism of terpenoids and polyketides	Carotenoid biosynthesis	1	ko00906
Metabolism	Energy metabolism	Nitrogen metabolism	1	ko00910
Metabolism	Lipid metabolism	Biosynthesis of unsaturated fatty acids	1	ko01040
Metabolism	Global and overview maps	Carbon metabolism	1	ko01200
Metabolism	Global and overview maps	Fatty acid metabolism	1	ko01212
Environmental Information Processing	Membrane transport	ABC transporters	1	ko02010
Environmental Information Processing	Signal transduction	MAPK signaling pathway - plant	1	ko04016
Cellular Processes	Transport and catabolism	Peroxisome	1	ko04146

Table S15. Network genes for the yellow module.

No.	Gene annotation	IC	GS	MM	Homologous protein GenBank ID	Protein name	Blast nr *p* value
1	(+)-pulegone reductase-like, partial	56.6806	0.7446	0.9682	ABR17262.1	unknown [*Picea sitchensis*]	8.48×10^{-113}
2	chalcone--flavonone isomerase 2	56.1381	0.6990	0.9746	ADE77594.1	unknown [*Picea sitchensis*]	0
3	chalcone synthase 2	55.5254	0.7063	0.9746	AEN84256.1	chalcone synthase, partial [*Picea sitchensis*]	0
4	digalactosyldiacylglycerol synthase 1, chloroplastic	53.7546	0.7881	0.9604	XP_002264659.1	PREDICTED: digalactosyldiacylglycerol synthase 1, chloroplastic [*Vitis vinifera*]	0
5	chalcone synthase 2	52.1909	0.6703	0.9467	CAA05214.1	chalcone synthase-like protein [*Pinus strobus*]	0
6	NADPH oxidoreductase, putative	51.8365	0.7582	0.9472	CAI56321.1	TPA: leucoanthocyanidin reductase [*Pinus taeda*]	0
7	chalcone synthase	51.7885	0.7330	0.9496	AEN84256.1	chalcone synthase, partial [Picea sitchensis]	5.24×10^{-129}
8	receptor-like protein kinase HSL1-like	51.4997	0.6073	0.9420	XP_012092411.1	PREDICTED: receptor-like protein kinase HSL1 [*Jatropha curcas*]	0
9	1-aminocyclopropane-1-carboxylate synthase	51.4890	0.6517	0.9420	ABM60748.1	ACC synthase [*Picea engelmannii* × *Picea glauca*]	0
10	pleiotropic drug resistance protein 1-like	51.2802	0.6587	0.9286	XP_010249930.1	PREDICTED: pleiotropic drug resistance protein 1-like [*Nelumbo nucifera*]	2.45×10^{-134}
11	chorismate mutase	51.1037	0.6780	0.9502	XP_006857227.2	PREDICTED: chorismate mutase 1, chloroplastic [*Amborella trichopoda*]	2.02×10^{-85}
12	Os09g0332600	50.9143	0.7011	0.9273	KVI05977.1	hypothetical protein Ccrd_015657 [*Cynara cardunculus* var. *scolymus*]	0
13	cinnamoyl-CoA reductase, putative	50.8942	0.6314	0.9298	AFC38436.1	cinnamoyl-CoA reductase [*Pinus radiata*]	4.04×10^{-161}
14	Leucoanthocyanidin dioxygenase family protein	49.3838	0.6814	0.9395	AHW42460.1	GA2ox10 [*Pinus tabuliformis*]	3.20×10^{-177}
15	chalcone synthase	48.2038	0.7420	0.9364	AGU43679.1	pinocembrin chalcone synthase [*Pinus massoniana*]	0
16	phospholipid-transporting ATPase 1	47.6266	0.7132	0.9166	XP_002271241.2	PREDICTED: phospholipid-transporting ATPase 1 [*Vitis vinifera*]	0
17	AMP dependent CoA ligase, putative	47.6191	0.5341	0.9382	AGX45528.1	4-coumarate:CoA ligase [*Pinus taeda*]	0
18	chalcone synthase 1-like	47.5932	0.6804	0.9432	ABK24577.1	unknown [*Picea sitchensis*]	0
19	dihydrolipoyllysine-residue succinyltransferase component of 2-	46.8763	0.6204	0.9375	ADE77401.1	unknown [*Picea sitchensis*]	0

	oxoglutarate dehydrogenase complex 1, mitochondrial						
20	oxalate--CoA ligase-like	46.4977	0.5413	0.9282	ABR16382.1	unknown [*Picea sitchensis*]	0
21	kinase, putative	46.3291	0.6360	0.9217	XP_006856200.1	PREDICTED: wall-associated receptor kinase-like 14 [*Amborella trichopoda*]	4.05×10^{-163}
22	not_annotated	46.2297	0.7762	0.9342	ACN40312.1	unknown [*Picea sitchensis*]	0
23	3-ketoacyl-CoA thiolase B, putative	45.4468	0.6005	0.9304	EMS60447.1	3-ketoacyl-CoA thiolase 2, peroxisomal [*Triticum urartu*]	1.67×10^{-93}
24	ATP-binding cassette transporter	45.2665	0.6962	0.8997	XP_010273226.1	PREDICTED: pleiotropic drug resistance protein 1-like [*Nelumbo nucifera*]	3.94×10^{-118}
25	probable voltage-gated potassium channel subunit beta-like	45.0376	0.5778	0.9425	ABK21055.1	unknown [*Picea sitchensis*]	0
26	trans-cinnamate 4-monooxygenase-like	45.0329	0.6360	0.9529	AAD23378.1	trans-cinnamate 4-hydroxylase [*Pinus taeda*]	0
27	leucoanthocyanidin dioxygenase family protein	44.9619	0.6793	0.9238	AHW42460.1	GA2ox10 [*Pinus tabuliformis*]	2.34×10^{-166}
28	pleiotropic drug resistance protein 1-like	44.2948	0.8078	0.9146	XP_010273226.1	PREDICTED: pleiotropic drug resistance protein 1-like [*Nelumbo nucifera*]	3.32×10^{-143}
29	not annotated	44.0103	0.7232	0.9318	AAP85250.1	phenylalanine ammonia-lyase [*Pinus pinaster*]	0
30	probable chalcone--flavonone isomerase 3	43.6128	0.6644	0.9295	ABK23299.1	unknown [*Picea sitchensis*]	1.91×10^{-95}
31	carboxylesterase np, putative	43.5740	0.6590	0.8968	XP_010266577.1	PREDICTED: uncharacterized protein LOC104604050 isoform X1 [*Nelumbo nucifera*]	5.78×10^{-128}
32	phospholipid/glycerol acyltransferase family protein	43.0202	0.6095	0.9293	ABR17313.1	unknown [*Picea sitchensis*]	0
33	Os09g0332600	42.9770	0.7291	0.9022	XP_010910610.1	PREDICTED: pleiotropic drug resistance protein 3-like [*Elaeis guineensis*]	0
34	proline-rich receptor-like protein kinase PERK13-like	42.4859	0.7509	0.9344	XP_011625141.1	PREDICTED: chitin elicitor receptor kinase 1 [*Amborella trichopoda*]	0
35	not_annotated	42.2507	0.7129	0.8830	XP_006855017.1	PREDICTED: F-box/LRR-repeat protein At1g67190 [*Amborella trichopoda*]	0

36	S-adenosylmethionine-dependent methyltransferase, putative	42.1960	0.6760	0.9190	CBI30090.3	unnamed protein product [*Vitis vinifera*]	1.55×10^{-131}
37	leucoanthocyanidin dioxygenase-like	41.9900	0.6905	0.9172	ABK24773.1	unknown [*Picea sitchensis*]	1.03×10^{-96}
38	leucoanthocyanidin dioxygenase family protein	41.3561	0.6974	0.9020	AHW42460.1	GA2ox10 [*Pinus tabuliformis*]	1.01×10^{-92}
39	uninformative	41.0187	0.7734	0.8943	B8LPG7.1	RecName: Full=Anamorsin homolog 2; AltName: Full=Fe-S cluster assembly protein DRE2 homolog 2	2.77×10^{-128}
40	probable mannitol dehydrogenase-like	40.8904	0.7782	0.8969	ABK26008.1	unknown [*Picea sitchensis*]	2.27×10^{-153}
41	not annotated	40.8380	0.6281	0.9360	ABK21911.1	unknown [*Picea sitchensis*]	0
42	transcription factor AtMYC2, putative	40.7092	0.7511	0.8872	ABR16623.1	unknown [*Picea sitchensis*]	0
43	leucoanthocyanidin dioxygenase	40.5531	0.6737	0.9123	AHW42460.1	GA2ox10 [*Pinus tabuliformis*]	3.19×10^{-141}
44	flavonoid 3',5'-hydroxylase 2	39.3982	0.6678	0.8965	ABR16821.1	unknown [*Picea sitchensis*]	0
45	oligopeptide transporter 7-like isoform X2	38.4851	0.6047	0.9400	XP_008806363.1	PREDICTED: oligopeptide transporter 7-like [*Phoenix dactylifera*]	0
46	ATP-citrate synthase beta chain protein 1-like	38.3733	0.5246	0.9003	ACN40574.1	unknown [*Picea sitchensis*]	0
47	malic enzyme, putative	37.9654	0.6547	0.9356	ABR18338.1	unknown [*Picea sitchensis*]	0
48	serine/threonine kinase, putative	37.9482	0.6445	0.9222	XP_006845584.1	PREDICTED: CBL-interacting serine/threonine-protein kinase 21 [*Amborella trichopoda*]	7.04×10^{-137}
49	protein TRANSPARENT TESTA 12	37.8402	0.6048	0.9098	AKC96391.1	multidrug and toxin compound extrusion protein [*Pinus radiata*]	0

50	anthocyanidin reductase	37.3895	0.5996	0.8979	ABR18365.1	unknown [*Picea sitchensis*]	9.10×10^{-159}
51	geraniol 8-hydroxylase-like	36.7439	0.6637	0.8869	ABK24553.1	unknown [*Picea sitchensis*]	0
52	leucoanthocyanidin dioxygenase isoform X1	36.6782	0.6136	0.9285	XP_009765337.1	PREDICTED: protein SRG1-like [*Nicotiana sylvestris*]	6.30×10^{-114}
53	FAD-dependent urate hydroxylase-like	36.4770	0.7773	0.8800	XP_006838554.1	PREDICTED: zeaxanthin epoxidase, chloroplastic [*Amborella trichopoda*]	5.61×10^{-145}
54	anthocyanidin reductase	36.0237	0.6102	0.8896	ABK24620.1	unknown [*Picea sitchensis*]	3.23×10^{-172}
55	not annotated	35.8647	0.7482	0.8819	ABK21817.1	unknown [*Picea sitchensis*]	3.60×10^{-69}
56	cytochrome P450 78A4-like	35.4014	0.7148	0.9228	AJP06264.1	CYP78A1 [*Pinus tabuliformis*]	0
57	not annotated	34.2710	0.5008	0.8891	ABR16367.1	unknown [*Picea sitchensis*]	7.40×10^{-177}
58	ocs element-binding factor 1	33.6663	0.6296	0.9095	AFG52929.1	hypothetical protein 0_7512_01, partial [*Pinus taeda*]	4.28×10^{-22}
59	aconitate hydratase, cytoplasmic-like	33.1805	0.7341	0.8498	XP_006452377.1	hypothetical protein CICLE_v10007338mg [*Citrus clementina*]	0
60	not annotated	33.1589	0.5065	0.9074	XP_010273789.1	PREDICTED: ATPase 10, plasma membrane-type-like isoform X1 [*Nelumbo nucifera*]	0
61	1,4-dihydroxy-2-naphthoyl-CoA thioesterase 1	33.0205	0.6913	0.9170	ABK24103.1	unknown [*Picea sitchensis*]	7.35×10^{-50}
62	leucoanthocyanidin dioxygenase	32.3650	0.6889	0.8607	AHW42460.1	GA2ox10 [*Pinus tabuliformis*]	1.70×10^{-163}
63	putative transcription factor bHLH041-like	31.7280	0.4585	0.8671	XP_004288955.2	PREDICTED: putative transcription factor bHLH041 [*Fragaria vesca* subsp. *vesca*]	2.29×10^{-36}

64	Helicase domain-containing protein / IBR domain-containing protein / zinc finger protein-related, putative isoform 1	31.6863	-0.7203	-0.8644	XP_010266797.1	PREDICTED: putative uncharacterized protein At4g01020, chloroplastic [*Nelumbo nucifera*]	0
65	transcription factor MYB12-like	31.2269	0.6855	0.8933	XP_009334363.1	PREDICTED: myb-related protein 308-like [*Pyrus × bretschneideri*]	$1.33×10^{-71}$
66	not annotated	31.2214	0.5857	0.8758	ACN41013.1	unknown [*Picea sitchensis*]	0
67	not annotated	30.9758	-0.8330	-0.8649	ABK22237.1	unknown [*Picea sitchensis*]	$1.70×10^{-99}$
68	aluminum activated malate transporter family protein	29.3594	0.6337	0.9012	XP_007039541.1	Aluminum-activated malate transporter 12 [*Theobroma cacao*]	$8.41×10^{-95}$
69	arogenate dehydratase/prephenate dehydratase 6, chloroplastic-like	28.9790	0.5940	0.9017	XP_010243735.1	PREDICTED: arogenate dehydratase/prephenate dehydratase 6, chloroplastic-like [*Nelumbo nucifera*]	$2.70×10^{-154}$
70	aluminum-activated malate transporter 12-like	28.2741	0.6435	0.8952	XP_008390303.1	PREDICTED: aluminum-activated malate transporter 10-like [*Malus domestica*]	$9.27×10^{-77}$
71	AP-2 complex subunit alpha-1	27.9390	0.5882	0.8486	XP_010278258.1	PREDICTED: AP-2 complex subunit alpha-1-like isoform X1 [*Nelumbo nucifera*]	0
72	phosphatidylinositol-4-phosphate 5-kinase isolog	27.2948	0.5995	0.8534	XP_002963939.1	hypothetical protein SELMODRAFT_230263 [*Selaginella moellendorffii*]	$4.13×10^{-109}$
73	not annotated	26.7664	0.6940	0.8806	ADE76959.1	unknown [*Picea sitchensis*]	3.97×10-^{105}
74	flavonol synthase/flavanone 3-hydroxylase	26.3068	0.6614	0.8736	XP_006844783.2	PREDICTED: protein SRG1 [*Amborella trichopoda*]	$2.80×10^{-105}$
75	probable terpene synthase 9	25.8606	0.4299	0.8418	Q84KL5.1	RecName: Full=Alpha-farnesene synthase [*Pinus taeda*]	0
76	carbamoyl-phosphate synthase large chain-like	25.3249	0.5319	0.8678	XP_009593636.1	PREDICTED: LOB domain-containing protein 1-like [*Nicotiana tomentosiformis*]	$1.26×10^{-41}$

77	dynamin, putative	25.3123	0.6278	0.8497	ABR18441.1	unknown [*Picea sitchensis*]	0
78	alcohol dehydrogenase-like	25.1284	0.6795	0.8554	ABK24305.1	unknown [*Picea sitchensis*]	0
79	uninformative	24.8370	0.4957	0.8442	ADE76466.1	unknown [*Picea sitchensis*]	4.58×10^{-79}
80	LOB domain-containing protein	24.3259	0.4804	0.8402	ABK26854.1	unknown [*Picea sitchensis*]	9.46×10^{-38}
81	alcohol dehydrogenase-like 3-like isoform X2	23.2495	0.6516	0.8559	ABK25905.1	unknown [*Picea sitchensis*]	1.72×10^{-168}
82	hypothetical protein	22.8781	0.6132	0.8649	XP_002990613.1	hypothetical protein SELMODRAFT_429046 [*Selaginella moellendorffii*]	1.92×10^{-10}
83	strictosidine synthase 3	20.2302	0.4458	0.8253	ABR18211.1	unknown [*Picea sitchensis*]	0
84	putative cinnamoyl CoA reductase	19.4196	0.6831	0.8114	ABK24762.1	unknown [*Picea sitchensis*]	1.76×10^{-162}
85	RING-H2 zinc finger protein RHA1a	19.4119	0.5481	0.8516	ABK24745.1	unknown [*Picea sitchensis*]	7.30×10^{-17}
86	uninformative	17.2187	0.5155	0.8274	ABR17654.1	unknown [*Picea sitchensis*]	0
87	anthocyanidin reductase	15.2639	0.3806	0.8140	ABR18365.1	unknown [*Picea sitchensis*]	4.19×10^{-169}
88	glycosyl hydrolase family 9 protein	13.3237	0.5817	0.7378	CAA52343.1	cellulase [*Sambucus nigra*]	4.15×10^{-175}
89	flavonoid 3',5'-hydroxylase 2	12.5318	0.5411	0.7257	ACN40555.1	unknown [*Picea sitchensis*]	0
90	ATP binding protein, putative	9.56913	0.5406	0.7461	XP_009418900.1	PREDICTED: putative receptor-like protein kinase At4g00960 [*Musa acuminata* subsp. *malaccensis*]	4.36×10^{-126}

91	salt tolerance protein-like	9.39871	0.6670	0.7601	ABK24665.1	unknown [*Picea sitchensis*]	1.58×10^{-129}
92	L-ascorbate oxidase	8.53832	0.3856	0.7203	ADE75758.1	unknown [*Picea sitchensis*]	0
93	lysine histidine transporter 1	6.15054	0.6016	0.6654	XP_009589743.1	PREDICTED: GABA transporter 1 [*Nicotiana tomentosiformis*]	1.17×10^{-142}

Table S16. KEGG enrichment result for genes with the top 30 highest GS values in the yellow module.

KEGG A class	KEGG B class	Pathway	Count (47 in total)	Pathway ID
Metabolism	Global and overview maps	Metabolic pathways	37	ko01100
Metabolism	Global and overview maps	Biosynthesis of secondary metabolites	32	ko01110
Metabolism	Biosynthesis of other secondary metabolites	Flavonoid biosynthesis	19	ko00941
Metabolism	Global and overview maps	Carbon metabolism	6	ko01200
Metabolism	Global and overview maps	Biosynthesis of amino acids	4	ko01230
Metabolism	Carbohydrate metabolism	Glycolysis / Gluconeogenesis	4	ko00010
Environmental Information Processing	Membrane transport	ABC transporters	3	ko02010
Organismal Systems	Environmental adaptation	Circadian rhythm - plant	3	ko04712
Cellular Processes	Transport and catabolism	Endocytosis	3	ko04144
Metabolism	Energy metabolism	Carbon fixation in photosynthetic organisms	2	ko00710
Metabolism	Carbohydrate metabolism	Citrate cycle (TCA cycle)	2	ko00020
Metabolism	Lipid metabolism	Fatty acid degradation	2	ko00071
Metabolism	Biosynthesis of other secondary metabolites	Flavone and flavonol biosynthesis	2	ko00944
Metabolism	Lipid metabolism	Glycerolipid metabolism	2	ko00561
Metabolism	Carbohydrate metabolism	Glyoxylate and dicarboxylate metabolism	2	ko00630
Environmental Information Processing	Signal transduction	MAPK signaling pathway - plant	2	ko04016
Metabolism	Amino acid metabolism	Phenylalanine, tyrosine and tryptophan biosynthesis	2	ko00400
Metabolism	Biosynthesis of other secondary metabolites	Phenylpropanoid biosynthesis	2	ko00940
Metabolism	Carbohydrate metabolism	Pyruvate metabolism	2	ko00620
Metabolism	Amino acid metabolism	Tyrosine metabolism	2	ko00350
Metabolism	Global and overview maps	2-Oxocarboxylic acid metabolism	1	ko01210
Metabolism	Lipid metabolism	alpha-Linolenic acid metabolism	1	ko00592
Metabolism	Carbohydrate metabolism	Ascorbate and aldarate metabolism	1	ko00053
Metabolism	Metabolism of terpenoids and polyketides	Carotenoid biosynthesis	1	ko00906
Metabolism	Amino acid metabolism	Cysteine and methionine metabolism	1	ko00270
Metabolism	Carbohydrate metabolism	Fructose and mannose metabolism	1	ko00051
Metabolism	Lipid metabolism	Glycerophospholipid metabolism	1	ko00564
Metabolism	Carbohydrate metabolism	Inositol phosphate metabolism	1	ko00562
Metabolism	Amino acid metabolism	Lysine degradation	1	ko00310
Metabolism	Energy metabolism	Nitrogen metabolism	1	ko00910
Metabolism	Energy metabolism	Oxidative phosphorylation	1	ko00190

Metabolism	Carbohydrate metabolism	Pentose phosphate pathway	1	ko00030
Environmental Information Processing	Signal transduction	Phosphatidylinositol signaling system	1	ko04070
Environmental Information Processing	Signal transduction	Plant hormone signal transduction	1	ko04075
Metabolism	Carbohydrate metabolism	Propanoate metabolism	1	ko00640
Genetic Information Processing	Transcription	Spliceosome	1	ko03040
Metabolism	Carbohydrate metabolism	Starch and sucrose metabolism	1	ko00500
Metabolism	Amino acid metabolism	Tryptophan metabolism	1	ko00380
Metabolism	Metabolism of cofactors and vitamins	Ubiquinone and other terpenoid-quinone biosynthesis	1	ko00130
Metabolism	Metabolism of cofactors and vitamins	Vitamin B6 metabolism	1	ko00750

Table S17. RT-qPCR primers used in this study.

Gene	Forward primer	Reverse primer
chalcone synthase1	GTGTGGCTAAAGACCTGGCA	ATCGGCTCCAACTATGACGG
chalcone synthase2	GTTCTCCGCCTCGCTAAAGA	TCCGCTCCCACAATAAGTGC
chalcone synthase3	CAACAACGCGTCGTCCTTTC	GATTCGTCGCCTGCTTCAAC
chalcone synthase4	CAAGCGGAGAAGGGTTGGAT	TCCGCGGACAGTAACAAGAC
chalcone synthase5	AGGCACATTCTGAGCGAACA	GACTATTGCAGAGGGACGCT
ubiquitin	CTGGCTCTACCGGAACCAAC	ACGGATGTTCTCCTGTGTGTC
